# Promising physiological traits associated with nitrogen use efficiency in rice under reduced N application

**DOI:** 10.3389/fpls.2023.1268739

**Published:** 2023-11-20

**Authors:** Bathula Srikanth, Desiraju Subrahmanyam, Durbha Sanjeeva Rao, Sadu Narender Reddy, Kallakuri Supriya, Puskur Raghuveer Rao, Kuchi Surekha, Raman Meenakshi Sundaram, Chirravuri Naga Neeraja

**Affiliations:** ^1^ ICAR-Indian Institute of Rice Research, Hyderabad, India; ^2^ Professor Jayashankar Telangana State Agricultural University, Hyderabad, India

**Keywords:** maximum quantum yield of PSII, actual quantum yield of PSII, electron transport rate, nitrogen use efficiency, nitrogen, rice

## Abstract

Higher grain yield in high-yielding rice varieties is mostly driven by nitrogen (N) fertilizer applied in abundant amounts leading to increased production cost and environmental pollution. This has fueled the studies on nitrogen use efficiency (NUE) to decrease the N fertilizer application in rice to the possible extent. NUE is a complex physiological trait controlled by multiple genes, but yet to be completely deciphered in rice. With an objective of identifying the promising physiological traits associated with NUE in rice, the performance of 14 rice genotypes was assessed at N0, N50, N100, and N150 for four (two wet and two dry) seasons using agro-morphological, grain yield, flag leaf traits, photosynthetic pigment content, flag leaf gas exchange traits, and chlorophyll fluorescence traits. Furthermore, the data were used to derive various NUE indices to identify the most appropriate indices useful to screen rice genotypes at N50. Results indicate that with the increase in N application, cumulative grain yield increased significantly up to N100 (5.02 t ha^−1^); however, the increment in grain yield was marginal at N150 (5.09 t ha^−1^). The mean reduction of grain yield was only 26.66% at N50 ranging from 15.0% to 34.2%. The significant finding of the study is the identification of flag leaf chlorophyll fluorescence traits (F_v_/F_m_, ΦPSII, ETR, and qP) and C_i_ associated with grain yield under N50, which can be used to screen N use efficient genotypes in rice under reduced N application. Out of nine NUE indices assessed, NUpE, NUtE, and NUE_yield_ were able to delineate the high-yielding genotypes at N50 and were useful to screen rice under reduced N conditions. Birupa emerged as one of the high yielders under N50, even though it is a moderate yielder at N100 and infers the possibility of cultivating some of the released rice varieties under reduced N inputs. The study indicates the possibility of the existence of promising genetic variability for grain yield under reduced N, the potential of flag leaf chlorophyll fluorescence, and gas exchange traits as physiological markers and best suitable NUE indices to be deployed in rice breeding programs.

## Introduction

Rice, a grain crop, is the prime source of food for more than half of the global population (Ogawa et al., 2016; Lee, 2021). Owing to the development of high-yielding cultivars, and the application of chemical fertilizers, rice production has been continuously improved during the last 50 years, keeping pace with the increasing global population (FAO, 2018). Nitrogen (N) is one of the key nutrient elements required for growth and development of rice. However, soil N content in agricultural land cannot sustain the higher yields of improved rice varieties. Hence, the application of N fertilizer has become unavoidable to enhance rice yield (Wang et al., 2022). However, most of the fertilizers applied in agriculture is the main source of environmental losses of reactive N compounds contributing to N pollution globally (Sutton et al., 2013) as well as in South Asia (Raghuram et al., 2021). Global N fertilizer consumption has already exceeded 110 million tons per year (Hu et al., 2023). The excess application of N fertilizers is more intense in China and India, which account for nearly 50% of the global rice production and consumption (Muthayya et al., 2014).

In India, from 1961 to 2013, the percentage N fertilizer application in total N input in production of cereal crops enhanced from 8%–10% to 71%–75% (Sapkota et al., 2023). As price of fertilizers are at record levels and may remain elevated, there has been a huge burden on the country’s economy. In parallel to the higher N fertilizer application, nitrogen use efficiency (NUE) has also been observed to be decreasing continuously and is evolving to be a major problem in restraining production of rice. In 2013, NUE was in the range of 20%–24% (except 32% for wheat) due to several-fold increase in the use of N fertilizers and imbalanced usage of fertilizer nutrients (Sapkota et al., 2023). In China, the average application rate of N fertilizer has reached 180 kg/hm^2^, which is 75% higher than the world average. However, the NUE is only 28%–35%, which is 15%–20% lower than that of the global average NUE (Liu et al., 2013; Han et al., 2015). It is projected that only 30% to 50% of the applied N fertilizer is utilized by rice (Ladha et al., 2020), thus resulting in enormous N leaching, and increased soil acidification and water eutrophication, leading to N-related environmental pollution, which is also a concern for climate change. In addition to the crop production practices targeting NUE, developing N efficient rice varieties to reduce the global climate change impacts should be one of the major research objectives (Neeraja et al., 2016). Reducing the cost of production and minimizing the environmental pollution through loss of N in field by using rice cultivars having higher NUE that can reduce the application of N fertilizers without a greater reduction in grain yield to feed the ever-increasing global population are need of the hour (Ciampitti and Vyn, 2011; Sharma and Bali, 2017).

India faces a dual challenge with N in terms of both food and the environment. On the one hand, India consumes 17 million tons of N fertilizer each year, which represents 14% of the global total. On the other hand, since the green revolution in 1970, the use of N fertilizer has increased at an annual rate of approximately 6% (Sutton et al., 2017). In 2022–2023, the Government of India allocated US$7.6 billion for urea subsidies. India loses US$10 billion worth of reactive N each year as fertilizer value. India is second only to China in terms of N production and consumption (Tewatia and Chanda, 2017). Studies on NUE in India began two to three decades ago in order to achieve sustainable agriculture (Abrol et al., 2007; Abrol et al., 2008; Galloway et al., 2008). As most of the genetic potential to enhance NUE lies unutilized in the germplasm of agricultural crops, focus should be on screening and utilizing them to improve NUE rather than for N-responsive yield alone (Metson et al., 2021). At the Indian Council of Agricultural Research-Indian Institute of Rice Research (ICAR-IIRR), screening of indigenous and exotic rice germplasm, varieties, landraces, and advanced breeding lines at various N levels was carried out as part of National Innovations on Climate Resilient Agriculture (NICRA), Newton-Bhabha Virtual Centre on Nitrogen Efficiency of Whole-cropping Systems for improved performance and resilience in agriculture (NEWS India-UK) and South Asian Nitrogen Hub (SANH) projects. Donors with promising performance at 50% of the recommended N level were identified and mapping populations were established (Subrahmanyam et al., 2019). Some promising breeding lines for NUE were tested under All India Coordinated Rice Improvement Project (AICRIP) across multiple locations from 2018 and identified for NUE in rice.

NUE can be enhanced agronomically up to a certain level, beyond which biological crop improvement alone can break the barrier for further improvement (Chakraborty and Raghuram, 2011). Based on this insight, research on the biological basis of N-response and NUE in diverse crops gained momentum (Raghuram and Sharma, 2019). Understanding physiological processes of the plant controlling N utilization under various N management practices is vital to improve NUE (Ciampitti and Vyn, 2011; Sharma and Bali, 2017). NUE is a multigenic quantitative trait, involving various N-responsive mechanisms that are yet to be fully characterized (Mandal et al., 2022). Earlier studies have documented the association between N application rate on crop photosynthetic traits, NUE, and yield (Makino et al., 2003; Paponov and Engels, 2003; Yang et al., 2010; Kong et al., 2016; Liu et al., 2019; Ochieng’ et al., 2021; Shah et al., 2021). Photosynthesis is the plant’s most crucial process for growth, biomass production, and yield (Chen et al., 2018). Two of the key traits to determine photosynthetic capacity are specific leaf area and leaf N content (Hikosaka, 2004; Poorter et al., 2009), which enhances chlorophyll content, enzyme content, and enzyme activity, and ultimately improves photosynthetic efficiency (Giersch and Robinson, 1987; Nasar et al., 2020; Noor Shah et al., 2021; Ochieng’ et al., 2021). Chlorophyll is highly sensitive to variations in the N content in the soil as a great part (70% of leaf N) of N was reported to part of the pigment’s composition (Paul, 1990; Kopsell et al., 2004; Fathi and Zeidali, 2021; Moenirad et al., 2021).

Photosynthetic rate (Pn) and photosynthetic nitrogen use efficiency (PNUE), which is the ratio of Pn to leaf N content, are the two primary attributes affecting the photosynthesis and nutrient utilization by plant leaves (Nasar et al., 2022). Furthermore, PNUE also reflects the N allocation and the overall photosynthesis of the plant (Zhong et al., 2019; Nasar et al., 2021). The greater the photosynthetic rate, the higher the PNUE and the leaf N utilization rate of the plant (Ghannoum et al., 2005). Therefore, studying the photosynthesis and PNUE of the plant is a crucial way to reveal its effect on NUE of the crops. The reduced quantum yield under N deficiency situations can be ascribed to the reduced photosynthetic capacity of the plant, which is due to the reduction of the production of key enzymes like Rubisco in the photosynthesis process (Qi et al., 2013). In contrast, sufficient N in the plant enhances quantum yield through enhancing leaf area index and photosynthetic electron transfer chain (Qi et al., 2013; Moenirad et al., 2021). Hence, plant breeding programs should emphasize on improving the N uptake, utilization, and remobilization of plant-available N (Laperche et al., 2006).

In rice, the relationship between leaf traits and NUE is yet to be characterized (Xin et al., 2022). Similarly, plant traits associated with N-efficient varieties have not been completely explored (Zhu et al., 2022). In order to identify the physiological traits associated with NUE in rice under reduced application, a set of 14 genotypes with varying yield potential were assessed under four graded levels of N (N0, N50, N100, and N150) for four seasons by deploying agro-morphological, grain yield, flag leaf traits, photosynthetic pigment content, flag leaf gas exchange traits, chlorophyll fluorescence traits, and NUE indices as criteria.

## Materials and methods

### Plant materials and seasons

Based on studies conducted earlier under NEWS project, genotypes with varying yield potential, *viz*., Anjali, Birupa, Daya, Heera, Indira, Nidhi, N22, Tella Hamsa, VL Dhan 209, Vasumati, IR64, GQ25, Varadhan, and MTU 1010, were selected for characterization of their physiological traits. Details of rice genotypes used in the study are given in **Table 1**. The trial was conducted at ICAR-IIRR farm during four seasons [Kharif-2020 (wet), Rabi-2021 (dry), Kharif-2021 (wet), and Rabi-2022 (dry)]. For the two wet seasons, seeds were sown in the month of June and seedlings were transplanted in the month of July. For the two dry seasons, seeds were sown in the month of December and seedlings were transplanted in the month of January. N was applied in the form of urea in three equal splits at the basal stage, maximum vegetative stage, and panicle initiation stage. Crop was cultivated by following the standard package of practices of crop production and crop protection.

**Table 1:** Details of rice genotypes included in the study.

S. No.	Name	Parentage	Year of release	Duration	Ecosystem
1.	Anjali (IET-16430)	PR-19-2 x RR-149-1129	2002	90-95	Rain fed Direct Seeded
2.	Birupa (IET-8620)	ADT-27 x IR-8 x Annapurna	1994	130-135	Irrigated Medium and Rain fed Lands
3.	Daya (OR-131-13-13)	Kumar x CR-57-49	1985	120-125	Irrigated Medium
4.	Heera (IET-10973)	CR-404-48 x Cr-289-1208	1989	65-68	Rainfed Upland
5.	Indira CR MUT587-4 (IET2412)	Tainan 3 mutant	1980	125	Irrigated
6.	Nidhi (IET-9994)	Sona x ARC-14529	1997	120-125	Irrigated Early
7	N22 (Nagina-22)	A selection from Rajbhog	1978	85-102	Promising germplasm identified with tolerance to biotic and abiotic stresses
8	Tella Hamsa	HR-12 x T(N)1	1975	110-115	Irrigated
9	IR64 (IET-9671)	IR-5857-33- 2-1 x IR-2061-465- 1-5-5	1991	115-120	Irrigated
10	GQ25 (INGR20001) Restorer line	(Samba Mahsuri/SC5126-3-2-4)	2011	130-135	Irrigated
11	Varadhan	Swarna x 9314)/BR 827-35	2008	125	Irrigated
12	MTU 1010 (IET-15644)	Krishnaveni x IR-64	2000	120	Irrigated Medium Lands
13	Vasumati (IET 15391 RP3135-17-12-8-8)	PR-109/Pakistani Basmati selection from local collection	2002	135	Irrigated
14	VL Dhan 209	Himdhan/K39 / VL Dhan 211	2006	160- 165	Rainfed

### Meteorological data

Important weather parameters recorded during the crop growing period is given in **Table 2**. During wet season 2020, mean maximum temperature was 30.7°C while mean minimum temperature was 21.9°C. The mean relative humidity was 93.4%, with a total rainfall of 1,375.6 mm and mean bright sunshine hours was 4.8 h day^−1^. During dry season 2021, mean maximum temperature was 32.4°C while mean minimum temperature was 16.1°C. The mean relative humidity was 87.5%, with a total rainfall of 16.8 mm and mean bright sunshine hours was 7.9 h day^−1^. During wet season 2021, mean maximum temperature was 30.7°C while mean minimum temperature was 22.6°C. The mean relative humidity was 93.9%, with a total rainfall of 823.8 mm and mean bright sunshine hours was 5.0 h day^−1^. During dry season 2022, mean maximum temperature was 32.5°C while mean minimum temperature was 17.3°C. The mean relative humidity was 84.4%, a total rainfall of 14.0 mm was received, and mean bright sunshine hours was 7.5 h day^−1^.

**Table 2:** Important weather parameters recorded during crop growing period at IIRR, Hyderabad.

Weather Parameter	2020-21		2021-22	
	Kharif (Wet)	Rabi (dry)	Kharif (Wet)	Rabi (dry)
Mean Max.Temperature (°C)	30.7	32.4	30.7	32.5
Mean Min.Temperature (°C)	21.9	16.1	22.6	17.3
Mean Relative Humidity (%)	93.4	87.5	93.9	84.4
Total Rainfall (mm)	1375.6	16.8	823.8	14.0
Mean Sunshine duration (h day^-1^)	4.8	7.9	5.0	7.5

### Soil analysis and experimental design

The experimental plot soil was clay in texture, slightly alkaline (pH 8.25), non-saline (EC - 0.76 dS/m), and medium in organic carbon content (0.53%). Soil available nitrogen was low (213 kg/ha) with high available phosphorus (92 kg/ha) and potassium (641 kg/ha). Experiments were arranged in a split-plot design with nitrogen application rates as the main plot and genotypes as the subplot with three replications. The size of each plot was 15 m^2^ (5.0 m long, 3.0 m wide, and 12 rows with a 25-cm row spacing). Four graded levels of N, *viz.*, N150 [150% recommended dose of N (RDN)—150 kg N ha^−1^], N100 (100% RDN—100 kg N ha^−1^), N50 (50% RDN—50 kg N ha^−1^) and N0 (0% RDN—0 kg N ha^−1^), were used.

### Morpho-physiological traits and grain yield

The number of days taken for 50% of plants to flower in each genotype and each treatment was noted as days to 50% flowering and was expressed in days. The number of days taken from sowing to physiological maturity was recorded and was expressed in days. The flag leaf traits along with SLA and SLW were measured from five randomly selected leaves per plot in three replications during 50% flowering stage (Kumar et al., 2021). Flag leaf length was measured from top to bottom of leaf and width was measured at the widest leaf part using ruler and flag leaf area was calculated using the formula given by (Quarrie and Jones, 1979).


Flag leaf area=Flag leaf length×Flag leaf width×0.75

Flag leaf thickness is measured using a digital caliper and expressed in millimeters (mm). The flag leaves were oven dried after measuring length, width, and thickness for 3 days at 80°C and flag leaf dry weight was recorded using an electronic balance (Sartorius, Germany). Specific leaf area (SLA) was calculated by dividing leaf area with leaf dry weight, employing the formula of Kvet (1971), and expressed in cm^2^ g^−1^. Specific leaf weight (SLW) was determined by dividing leaf dry weight with leaf area, using the formula of Pearce (1968) and expressed in mg cm^−2^.

At physiological maturity, plots of 1 m^2^ area were harvested and threshed grain weight was determined after drying to 14% moisture content and converted to t ha^−1^ and straw weight was also recorded for the same (Kumar et al., 2021). Total dry matter was calculated as sum of the dry weights of the plant components and converted to t ha^−1^ (Amanullah and Inamullah, 2016).

### Photosynthetic pigment content

For the quantitative determination of leaf chlorophyll content, at 50% flowering stage, five plants were randomly chosen in each plot and the flag leaf was labeled to investigate gas exchange traits and photosynthetic pigment content. For the determination of pigment content, leaf tissue of each sample was cut into small pieces with a sharp razor blade and 25 mg of cut leaf pieces was placed into 10-mL tubes containing 10 mL of 80% acetone and stored in the dark for 48 h to ensure complete extraction of leaf chlorophyll pigment. The absorbance of the chlorophyll solution was measured by using a UV-VIS double beam spectrophotometer (Evolution 201, Thermo Scientific, USA). Chlorophyll a, chlorophyll b, and carotenoids were measured at 663.2, 646.8, and 470.0 nm, respectively, and expressed in mg g^−1^ fresh weight (fw). The content of chlorophyll a, chlorophyll b, total chlorophyll, and carotenoids were calculated as per the formulas given by Lichtenthaler and Wellburn (1983).

### Flag leaf gas exchange traits

Gas exchange traits in flag leaf such as photosynthetic rate (Pn), stomatal conductance (g_s_), transpiration rate (E), and internal CO_2_ concentration (C_i_) were recorded at 50% flowering stage by using the Infra-Red Gas Analyzer portable photosynthesis measurement system (6400XT, LICOR, USA) attached to a leaf chamber fluorometer, which was used as the light source. During measurements, the photosynthetically active radiation (PAR) was kept at 1,200 µmol m^−2^ s^−1^. The CO_2_ concentration was maintained at 387 ± 6 ppm. These measurements were made between 10:00 a.m. and 12.00 noon at all the sampling dates. Pn was expressed in μmol (CO_2_) m^−2^ s^−1^, g_s_ was expressed in mol (H_2_O) m^−2^ s^−1^, E was expressed in mmol (H_2_O) m^−2^ s^−1^, and C_i_ was expressed in ppm. PNUE was calculated as given by Ye et al. (2019a).

### Chlorophyll fluorescence characteristics

Chlorophyll fluorescence traits were measured with MINI PAM-II Photosynthesis Yield Analyzer (Heinz Walz GmbH, Germany) during 50% flowering stage. The instrument was connected to a desktop PC with WinControl-3 software. The flag leaves were dark-adapted for 30 min before recording fluorescence traits and the following fluorescence traits were calculated: the maximum quantum yield of PSII (F_v_/F_m_), actual quantum yield of PSII (ΦPSII), electron transport rate (ETR), coefficient of photochemical quenching (qP), and coefficient of non-photochemical quenching (qN) (Maxwell and Johnson, 2000).

### Nitrogen content estimation and NUE indices

Flag leaf samples collected at 50% flowering stage were used to determine flag leaf N content, and grain and straw samples were collected from 1 m^2^ area at harvest. Samples were dried under shade and then in hot air oven at 60°C. Oven-dried samples were ground to fine powder using a grinder and stored in butter paper covers for estimating N concentration. The samples were digested in sulfuric acid (H_2_SO_4_) using block digestion unit and analyzed for their total N content by the micro Kjeldahl distillation method using automatic N analyzer (Kjeltec 8400 Analyzer FOSS, Denmark) with steam distillation and the N content was expressed as percentage. NUE indices such as nitrogen uptake efficiency (NUpE), nitrogen utilization efficiency (NUtE), nitrogen use efficiency_yield_ (NUE_yield_), agronomic efficiency (AE), physiology efficiency (PE), partial factor productivity (PFP), apparent nitrogen recovery efficiency (ANRE), and nitrogen harvest index (NHI) were calculated as per formulas given in Congreves et al. (2021) and nitrogen utilization index (NUI) was calculated as per the formula given in Huang et al. (2018).


$$NUpE=\frac{Plant\;N}{Fertilizer\;N+Soil\;N}\;x\;100$$

$$NUtE=\frac{Yield}{Plant\;N}$$

$$NUE_{yield}=NUpE\;x\;NUtE$$

$$AE=\frac{Yield_{f}-Yield_{0}}{Fertilizer\;N}$$

$$PE=\frac{Yield_{f}-Yield_{0}}{Plant\;N_{f}-Plant\;N_{0}}$$

$$PFP=\frac{Yield_{f}}{Fertilizer\;N}$$

$$ANRE=\frac{Plant\;N_{f}-Plant\;N_{0}}{Fertilizer\;N}\;x\;100$$

$$NHI=\frac{Yield\;N}{Plant\;N}\;x\;100$$

$$NUI=\frac{Total\;dry\;matter}{Plant\;N}$$

where Plant N is the amount of N in a plant, Yield N is the amount of grain N in a plant, Plant N_f_ is the amount of N in a fertilized plant, Plant N_0_ is the amount of N in a non-fertilized plant, Yield_f_ is the grain yield of a fertilized plant, and Yield_0_ is the grain yield of a non-fertilized plant.

### Statistical analysis

Two-way analysis of variance (ANOVA) was performed using an open source software R (R Core Team, 2012) with Agricolae package (de Mendiburu, 2012). Statistical significance of the parameters means was determined by performing Fisher’s LSD test to test the statistical significance.

## Results

ANOVA indicates that 29 morpho-physiological traits including grain yield and N uptake traits noted significant variation with treatment and among the genotypes (**Table 3**). Interaction between treatment and genotypes was significant except for E, chlorophyll a, total chlorophyll, and carotenoids. Season × genotypes was significant except for flag leaf thickness, SLA, SLW, and F_v_/F_m_. Season × treatment was significant for Pn, g_s_, E, days to 50% flowering, days to physiological maturity, chlorophyll a, total chlorophyll, carotenoids, PNUE, total dry matter, grain N uptake, straw N uptake, and total N uptake. Season × treatment × genotypes was significant for g_s_, E, days to physiological maturity, and photosynthetic pigments. Among the NUE indices, ANOVA showed significant effect of treatment and variation among genotypes and interaction between treatment and genotypes. Three indices (NUtE, NUE_yield_, and NUI) noted significant interaction for season × treatment and six indices (ANRE, NUpE, NUtE, NUE_yield_, NUI, and NHI) noted significant interaction for season × genotypes. Significant interaction was not observed in any of the indices for season × treatment × genotypes.

Table 3ANOVA for morpho-physiological parameters, grain yield and nitrogen use efficiency indices.

**Table:** 

		Grain Yield			Total dry matter			Days to 50% Flowering			Days to maturity			Flag leaf length			Flag leaf width					
EFFECT	Df	Mean Sq	F value	Pr(>F)	Mean Sq	F value	Pr(>F)	Mean Sq	F value	Pr(>F)	Mean Sq	F value	Pr(>F)	Mean Sq	F value	Pr(>F)	Mean Sq		F value		Pr(>F)	
Season (S)	3	1.381			8.982			779.26			351.34			150.23			0.703					
Rep x Season	6	0.054			0.369			0.34			0.43			1.2			0.002					
Treatment (T)	3	204.141	1479.63	0.0000**	451.062	185.28	0.0000**	670.04	392.33	0.0000**	677.41	104.74	0.0000**	2527.06	1152.8	0.0000**	2.225		688.36		0.0000**	
S x T	9	0.138	1.95	0.0922	2.434	9.86	0.0000**	1.71	2.37	0.0442*	6.47	3.28	0.0095**	2.19	2.14	0.066	0.003		1.47		0.2163	
Error (T)	24	0.071			0.247			0.72			1.97			1.02			0.002					
Genotypes (G)	13	15.432	73.51	0.0000**	56.804	49.06	0.0000**	4420.04	90.3	0.0000**	2951.57	41.17	0.0000**	342.23	46.44	0.0000**	0.295		15.22		0.0000**	
S x G	39	0.21	2.46	0.0000**	1.158	3.56	0.0000**	48.61	53.35	0.0000**	71.69	53.78	0.0000**	7.37	2.58	0.0000**	0.019		1.69		0.0074**	
T x G	39	0.83	13.37	0.0000**	3.146	11.26	0.0000**	1.42	2.51	0.0001**	2.73	1.57	0.0333*	13.12	12.06	0.0000**	0.014		3		0.0000**	
S x T x G	117	0.062	0.73	0.9802	0.279	0.86	0.8379	0.56	0.62	0.9989	1.73	1.3	0.0332*	1.09	0.38	1	0.005		0.41		1	
Residual	416	0.085			0.325			0.91			1.33			2.86			0.011					
Total	671	1.348			3.707			95.74			67.4			21.86			0.029					
CV (%)		6.41			5.27			0.86			1.1			3.28			3.31

**Table:** 

		Flag leaf area			Flag leaf thickness			Flag leaf dry weight			Specific leaf area			Specific leaf weight			Chlorophyll a content					
EFFECT	Df	Mean Sq	F value	Pr(>F)	Mean Sq	F value	Pr(>F)	Mean Sq	F value	Pr(>F)	Mean Sq	F value	Pr(>F)	Mean Sq	F value	Pr(>F)	Mean Sq		F value		Pr(>F)	
Season (S)	3	524.51			0.0074			0.00786			1177.8			0.414			16.367					
Rep x Season	6	3.08			0.0003			0.00002			50.7			0.022			0.019					
Treatment (T)	3	7787.13	2133.39	0.0000**	0.2696	1069.59	0.0000**	0.07705	1826.99	0.0000**	31447.6	249.38	0.0000**	12.431	270.42	0.0000**	46.847		248.79		0.0000**	
S x T	9	3.65	2.12	0.0687	0.0003	0.8	0.6211	0.00004	1.07	0.4177	126.1	2.04	0.0787	0.046	1.7	0.1449	0.188		4.38		0.0018**	
Error (T)	24	1.72			0.0003			0.00004			61.8			0.027			0.043					
Genotypes (G)	13	767.81	31.48	0.0000**	0.02	278.07	0.0000**	0.01483	47.29	0.0000**	244.7	2.49	0.0141*	0.095	2.58	0.0111*	0.907		6.9		0.0000**	
S x G	39	24.39	2.39	0.0000**	0.0001	0.41	0.9994	0.00031	2.12	0.0002**	98.4	1.24	0.1572	0.037	1.16	0.2377	0.132		3.31		0.0000**	
T x G	39	25.25	5.55	0.0000**	0.0009	4.32	0.0000**	0.0004	7.34	0.0000**	229.8	3.72	0.0000**	0.091	3.9	0.0000**	0.077		1.41		0.0837	
S x T x G	117	4.55	0.45	1	0.0002	1.16	0.1455	0.00005	0.37	1	61.7	0.78	0.9469	0.023	0.74	0.9761	0.054		1.37		0.0136*	
Residual	416	10.19			0.0002			0.00015			79.2			0.032			0.04					
Total	671	62.18			0.0018			0.00081			234.2			0.092			0.351					
CV (%)		3.96			5.43			4.28			3.5			3.68			8.81

**Table:** 

		Chlorophyll b content			Total chlorophyll content			Carotenoid content			Photosynthetic rate			Stomatal conductance			Transpiration rate					
EFFECT	Df	Mean Sq	F value	Pr(>F)	Mean Sq	F value	Pr(>F)	Mean Sq	F value	Pr(>F)	Mean Sq	F value	Pr(>F)	Mean Sq	F value	Pr(>F)	Mean Sq		F value		Pr(>F)	
Season (S)	3	2.414			30.508			1.305			112.17			0.2295			16.14					
Rep x Season	6	0.009			0.052			0.002			0.83			0.0026			0.19					
Treatment (T)	3	6.379	357.74	0.0000**	87.79	373.5	0.0000**	2.799	111.1	0.0000**	3233.33	260.81	0.0000**	6.088	135.99	0.0000**	401.41		148.61		0.0000**	
S x T	9	0.018	1.74	0.1345	0.235	2.79	0.0216*	0.025	4.86	0.0009**	12.4	9.36	0.0000**	0.0448	10.35	0.0000**	2.7		6.67		0.0000**	
Error (T)	24	0.01			0.084			0.005			1.33			0.0043			0.4					
Genotypes (G)	13	0.176	6.24	0.0000**	1.738	7.97	0.0000**	0.062	2.91	0.0049**	33.89	5.84	0.0000**	0.0576	2.83	0.0060**	5.43		2.33		0.0207*	
S x G	39	0.028	3.54	0.0000**	0.218	3.06	0.0000**	0.021	4.99	0.0000**	5.8	3.82	0.0000**	0.0204	3.94	0.0000**	2.33		3.83		0.0000**	
T x G	39	0.03	1.53	0.0439*	0.14	1.45	0.06835	0.012	1.35	0.1108	2.67	1.53	0.0429*	0.0125	1.6	0.0282*	1.03		1.31		0.1352	
S x T x G	117	0.02	2.47	0.0000**	0.097	1.36	0.0158*	0.009	2.16	0.0000**	1.75	1.15	0.1632	0.0078	1.5	0.0019**	0.79		1.29		0.0349*	
Residual	416	0.008			0.071			0.004			1.52			0.0052			0.61					
Total	671	0.055			0.651			0.026			17.58			0.0366			2.74					
CV (%)		14.13			9.45			10.71			5.48			12.63			9.29

**Table:** 

		Internal CO_2_ concentration			Flag leaf N content			Photosynthetic NUE			Maximum quantum yield of PSII			Actual quantum yield of PSII			Electron transport rate					
EFFECT	Df	Mean Sq	F value	Pr(>F)	Mean Sq	F value	Pr(>F)	Mean Sq	F value	Pr(>F)	Mean Sq	F value	Pr(>F)	Mean Sq	F value	Pr(>F)	Mean Sq		F value		Pr(>F)	
Season (S)	3	3014.2			4.685			423.85			0.01104			0.1293			96.24					
Rep x Season	6	74.6			0.014			1.19			0.00006			0.0014			2.78					
Treatment (T)	3	17442.3	38.61	0.0000**	18.603	90.53	0.0000**	1170	73.9	0.0000**	0.02683	372.77	0.0000**	0.2283	227.85	0.0000**	1259.42		276.28		0.0000**	
S x T	9	451.8	0.72	0.6887	0.205	5.66	0.0003**	15.83	4.88	0.0009**	0.00007	0.86	0.5706	0.001	0.73	0.6801	4.56		1.34		0.2672	
Error (T)	24	630.2			0.036			3.24			0.00008			0.0014			3.39					
Genotypes (G)	13	4995.8	2.45	0.0153*	1.142	3.58	0.0010**	81.2	3.4	0.0015**	0.00097	9.27	0.0000**	0.0056	2.45	0.0156*	19.07		1.75		0.0892	
S x G	39	2036.8	3.14	0.0000**	0.32	7.02	0.0000**	23.85	5.37	0.0000**	0.00011	0.84	0.7385	0.0023	2.14	0.0001**	10.92		2.85		0.0000**	
T x G	39	723.8	1.61	0.0269*	0.035	2.12	0.0011**	4.77	1.77	0.0101*	0.00016	4.01	0.0000**	0.0011	2.57	0.0000**	5.04		3.29		0.0000**	
S x T x G	117	449.4	0.69	0.9911	0.016	0.36	1	2.69	0.61	0.9993	0.00004	0.33	1	0.0004	0.38	1	1.54		0.4		1	
Residual	416	649.4			0.046			4.44			0.00012			0.0011			3.83					
Total	671	868.4			0.182			13.94			0.00029			0.0027			10.22					
CV (%)		9.42			7.47			9.65			1.13			10.55			7.43

**Table:** 

		Coefficient of photochemical quenching			Coefficient of non- photochemical quenching			Grain N Uptake			Straw N Uptake			Total N Uptake			Agronomic efficiency			Physiological efficiency		
EFFECT	Df	Mean Sq	F value	Pr(>F)	Mean Sq	F value	Pr(>F)	Mean Sq	F value	Pr(>F)	Mean Sq	F value	Pr(>F)	Mean Sq	F value	Pr(>F)	Mean Sq	F value	Pr(>F)	Mean Sq	F value	Pr(>F)
Season (S)	3	0.0220			0.0546			1415.0			161.1			1540.9			103.26			2319.5		
Rep x Season	6	0.0009			0.0008			28.0			18.6			66.8			11.14			212.9		
Treatment (T)	3	0.5712	89.75	0.0000**	0.1891	163.98	0.0000**	56077.3	655.70	0.0000**	9446.8	236.09	0.0000**	111305.3	860.55	0.0000**	2148.02	151.60	0.0000**	1122.9	34.30	0.0005**
S x T	9	0.0064	2.03	0.0807	0.0012	0.65	0.7474	85.5	2.87	0.0189*	40.0	3.30	0.0093**	129.3	2.51	0.0349*	14.17	1.54	0.2292	32.7	0.18	0.9787
Error (T)	24	0.0031			0.0018			29.8			12.1			51.6			9.22			183.2		
Genotypes (G)	13	0.0161	2.33	0.0210*	0.0080	2.97	0.0043**	2764.9	27.74	0.0000**	558.2	8.10	0.0000**	5508.5	21.31	0.0000**	744.46	40.63	0.0000**	511.1	3.98	0.0004**
S x G	39	0.0069	2.63	0.0000**	0.0027	1.98	0.0006**	99.7	3.31	0.0000**	68.9	5.32	0.0000**	258.5	4.74	0.0000**	18.32	1.05	0.3879	128.3	0.81	0.7792
T x G	39	0.0019	1.64	0.0231*	0.0012	3.05	0.0000**	168.7	10.38	0.0000**	62.2	5.69	0.0000**	350.9	8.73	0.0000**	126.98	14.52	0.0000**	98.9	2.56	0.0008**
S x T x G	117	0.0012	0.44	1	0.0004	0.30	1	16.3	0.54	0.9999	10.9	0.84	0.8654	40.2	0.74	0.9756	8.74	0.50	0.9998	38.7	0.25	1
Residual	416	0.0026			0.0014			30.1			13.0			54.5			17.38			157.7		
Total	671	0.0055			0.0025			350.2			72.6			692.0			49.21			160.4		
CV (%)		9.91			11.05			10.42			10.39			8.36			16.83			30.40

**Table:** 

		Partial factor productivity			Apparent nitrogen recovery efficiency			Nitrogen Uptake Efficiency			Nitrogen Utilization Efficiency			Nitrogen Use Efficiency			Nitrogen Utilization Index			Nitrogen Harvest Index		
EFFECT	Df	Mean Sq	F value	Pr(>F)	Mean Sq	F value	Pr(>F)	Mean Sq	F value	Pr(>F)	Mean Sq	F value	Pr(>F)	Mean Sq	F value	Pr(>F)	Mean Sq	F value	Pr(>F)	Mean Sq	F value	Pr(>F)
Season (S)	3	201.15			464.5			192.30			847.09			17.33			5073.5			0.0400		
Rep x Season	6	4.53			112.0			4.85			8.19			0.48			70.4			0.0007		
Treatment (T)	3	65881.17	2243.47	0.0000**	6666.6	81.23	0.0000**	454.84	38.28	0.0000**	776.85	34.69	0.0000**	144.09	54.35	0.0000**	46627.2	116.81	0.0000**	0.2128	156.81	0.0000**
S x T	9	29.37	2.19	0.0979	82.1	1.43	0.2639	11.88	1.62	0.1643	22.40	3.73	0.0047**	2.65	2.36	0.0450*	399.2	11.40	0.0000**	0.0014	2.23	0.0568
Error (T)	24	13.38			57.4			7.31			6.00			1.12			35.0			0.0006		
Genotypes (G)	13	1867.45	96.68	0.0000**	3434.8	15.04	0.0000**	682.03	22.54	0.0000**	221.28	4.14	0.0003**	189.68	76.20	0.0000**	1068.1	4.71	0.0001**	0.0184	6.87	0.0000**
S x G	39	19.32	1.27	0.1414	228.3	1.81	0.0034**	30.26	4.81	0.0000**	53.46	6.06	0.0000**	2.49	2.48	0.0000**	226.8	4.71	0.0000**	0.0027	3.13	0.0000**
T x G	39	155.53	21.12	0.0000**	351.3	6.69	0.0000**	47.42	10.65	0.0000**	23.30	5.98	0.0000**	9.94	14.78	0.0000**	74.0	3.47	0.0000**	0.0023	6.22	0.0000**
S x T x G	117	7.36	0.48	0.9999	52.5	0.42	0.9999	4.45	0.71	0.9871	3.90	0.44	1	0.67	0.67	0.9949	21.4	0.44	1	0.0004	0.43	1
Residual	416	15.25			126.5			6.29			8.82			1.00			48.1			0.0009		
Total	671	332.55			244.7			25.80			22.75			5.95			310.1			0.0024		
CV (%)		6.97			18.33			9.08			5.01			7.31			5.18			4.11		

### Cumulative data of four seasons

#### Morpho-physiological traits and grain yield

The genotype-wise values of all the measured traits of the study are presented in **Tables 4**–**7**. The range and mean values of morpho-physiological traits along with grain yield and NUE indices at various grades of N fertilizer application are presented in **Table 8**. Mean grain yield significantly increased from 2.82 to 5.09 t ha^−1^ and total dry matter significantly increased from 7.43 to 10.89 t ha^−1^ with the increase in N fertilizer application among the treatments (from N0 to N50, N100, and N150). Among the genotypes, Vasumati at N50 and MTU 1010 at N100 recorded the highest grain yield (4.22 and 5.84 t ha^−1^), while N22 recorded the lowest (2.46 and 3.28 t ha^−1^) at N50 and N100. The highest total dry matter was recorded in Varadhan (9.87 and 12.08 t ha^−1^) whereas N22 recorded the lowest (6.29 and 7.37 t ha^−1^) at N50 and N100. With increased N application from N0 to N150, mean days to 50% flowering and physiological maturity significantly increased from 96 to 101 days and 125 to 130 days. Among the genotypes, days to 50% flowering ranged from 83 (Anjali) to 111 days (Birupa) at N50 and 84 (Anjali) to 112 days (Birupa) at N100. Days to maturity ranged from 114 (Anjali) to 137 days (Daya) at N50 and 114 (Anjali) to 138 days (Birupa) at N100.

**Table 4:** Cumulative mean values of morpho-physiological traits along with grain yield and NUE indices at N0 in different genotypes.

Genotype	Anjali	Birupa	Daya	Heera	Indira	Nidhi	N22	Tella Hamsa	V L Dhan 209	Vasumati	IR64	GQ25	Varadhan	MTU 1010	Mean
**GY**	2.54	2.91	2.72	3.34	2.98	2.39	1.88	2.30	3.37	2.86	3.36	2.63	2.75	3.43	2.82
**TDM**	6.96	7.74	7.10	8.49	7.67	6.46	5.29	6.41	8.64	7.44	8.56	6.98	7.17	9.18	7.43
**DFF**	81	109	108	81	102	100	87	84	99	107	99	99	93	92	96
**DPM**	112	136	135	115	129	128	118	115	129	134	130	129	125	123	125
**FLL**	26.8	28.8	25.9	24.4	31.0	25.1	26.2	23.7	28.8	26.0	23.8	25.3	28.1	24.6	26.3
**FLW**	1.29	1.28	1.30	1.44	1.36	1.28	1.21	1.19	1.38	1.21	1.16	1.34	1.24	1.26	1.28
**FLA**	26.1	27.5	25.1	26.5	31.6	24.1	23.7	21.2	29.8	23.7	20.8	25.4	26.3	23.3	25.4
**FLT**	0.268	0.329	0.277	0.297	0.271	0.257	0.298	0.246	0.295	0.286	0.311	0.238	0.260	0.301	0.281
**FLDW**	0.123	0.129	0.120	0.127	0.154	0.118	0.119	0.102	0.141	0.113	0.098	0.122	0.124	0.108	0.121
**SLA**	212.1	213.0	208.6	208.9	205.5	203.3	198.2	207.4	212.1	210.0	212.4	208.4	210.7	215.3	209.0
**SLW**	4.72	4.70	4.80	4.80	4.87	4.92	5.05	4.84	4.72	4.77	4.73	4.80	4.75	4.66	4.80
**CHLa**	1.53	1.74	1.74	1.84	1.69	1.59	1.59	1.55	1.69	1.82	1.81	1.71	1.82	1.89	1.71
**CHLb**	0.428	0.511	0.478	0.447	0.486	0.499	0.370	0.421	0.513	0.503	0.453	0.502	0.552	0.527	0.478
**TCHL**	1.96	2.25	2.22	2.28	2.17	2.09	1.96	1.97	2.20	2.32	2.26	2.22	2.37	2.41	2.19
**CAR**	0.462	0.511	0.508	0.582	0.514	0.484	0.517	0.457	0.481	0.532	0.547	0.489	0.512	0.544	0.510
**Pn**	15.1	16.4	15.2	15.9	15.4	14.6	14.4	14.6	16.3	16.3	15.9	16.1	16.3	16.7	15.7
**gs**	0.277	0.322	0.272	0.296	0.285	0.246	0.238	0.248	0.292	0.287	0.256	0.295	0.306	0.317	0.281
**E**	4.64	5.07	4.72	4.94	4.91	4.65	4.15	4.96	5.00	5.39	5.08	4.90	5.50	5.28	4.94
**Ci**	287.9	288.9	287.7	273.0	275.2	269.0	263.1	273.4	282.7	282.4	275.5	280.2	289.5	306.4	281.1
**FLN**	2.29	2.01	1.92	2.25	1.98	2.04	1.95	2.32	2.00	2.09	2.35	2.15	2.15	2.08	2.11
**PNUE**	14.1	17.5	16.9	15.0	16.4	14.9	14.8	13.3	17.6	16.6	14.6	15.8	16.1	17.4	15.8
**F_v_/F_m_**	0.792	0.799	0.786	0.796	0.792	0.791	0.780	0.781	0.789	0.799	0.794	0.795	0.794	0.807	0.792
**ϕPSII**	0.320	0.318	0.288	0.305	0.303	0.292	0.306	0.319	0.297	0.313	0.319	0.298	0.316	0.338	0.309
**ETR**	22.7	21.2	20.5	21.4	21.1	20.3	21.6	21.9	21.2	21.7	22.1	20.5	22.4	23.2	21.5
**qP**	0.513	0.496	0.474	0.497	0.487	0.484	0.489	0.508	0.498	0.512	0.514	0.488	0.517	0.505	0.499
**qN**	0.425	0.408	0.414	0.443	0.391	0.464	0.435	0.438	0.422	0.424	0.407	0.447	0.436	0.410	0.426
**GNU**	27.6	30.5	27.7	38.1	31.1	26.1	20.3	24.6	36.5	30.8	35.3	28.7	29.5	41.4	30.6
**SNU**	24.3	21.9	22.4	26.5	22.6	22.2	17.9	25.6	28.7	20.0	30.9	24.2	22.2	34.3	24.5
**TNU**	51.9	52.4	50.1	64.6	53.8	48.2	38.2	50.2	65.2	50.8	66.2	52.9	51.7	75.7	55.1
**NUpE**	27.1	27.4	26.2	33.7	28.1	25.2	19.9	26.2	34.0	26.5	34.6	27.6	27.0	39.5	28.8
**NUtE**	49.3	55.7	54.8	52.0	56.0	49.8	49.4	45.7	52.0	56.4	51.0	49.9	53.1	45.3	51.5
**NUE_yield_**	13.3	15.2	14.2	17.5	15.5	12.5	9.8	12.0	17.6	14.9	17.5	13.7	14.3	17.9	14.7
**NUI**	134.9	148.3	142.9	131.9	143.9	134.6	138.7	128.0	133.3	146.6	130.0	132.3	138.9	121.3	136.1
**NHI**	53.4	58.5	55.5	59.0	57.9	53.9	53.0	49.0	55.9	60.6	53.4	54.3	57.1	54.7	55.4

Where, GY, Grain yield (t ha^-1^); TDM, Total dry matter (t ha^-1^); DFF, Days to 50% flowering; DPM, Days to physiological maturity; FLL, Flag leaf length (cm); FLW, Flag leaf width (cm); FLA, Flag leaf area (cm^2^); FLT, Flag leaf thickness (mm); FLDW, Flag leaf dry weight (g); SLA, Specific leaf area (cm^2^ g^-1^); SLW, Specific leaf weight (mg cm^-2^); CHLa, Chlorophyll a (mg g^-1^ fw); CHLb, Chlorophyll b (mg g^-1^ fw); TCHL, Total chlorophyll (mg g^-1^ fw); CAR, Carotenoids (mg g^-1^ fw); Pn, Photosynthetic rate (µmol [CO_2_] m^-2^ s^-1^); gs, Stomatal conductance (mol [H_2_O] m^-2^ s^-1^); E, Transpiration rate (mmol [H_2_O] m^-2^ s^-1^); Ci, Internal CO_2_ concentration (ppm); FLN, Flag leaf N content (%); PNUE, Photosynthetic nitrogen use efficiency (μmol [CO_2_] g^-1^ [N] s^-1^); F_v_/F_m_, Maximum quantum yield of PSII; ΦPSII, Actual quantum yield of PSII; ETR, Electron transport rate; qP, Coefficient of photochemical quenching; qN, Coefficient of non-photochemical quenching; GNU, Grain N uptake (kg N ha^-1^); SNU, Straw N uptake (kg N ha^-1^); TNU, Total plant N uptake (kg N ha^-1^); NUpE, Nitrogen uptake efficiency; NUtE, Nitrogen utilization efficiency; NUE_yield_, Nitrogen use efficiency_yield_; NUI, Nitrogen utilization index; NHI,Nitrogen harvest index.

**Table 5:** Cumulative mean values of morpho-physiological traits along with grain yield and NUE indices at N50 in different genotypes.

Genotype	Anjali	Birupa	Daya	Heera	Indira	Nidhi	N22	Tella Hamsa	V L Dhan 209	Vasumati	IR64	GQ25	Varadhan	MTU 1010	Mean
**GY**	3.18	4.08	3.49	3.92	4.04	3.42	2.46	2.96	3.71	4.22	3.74	3.79	4.20	4.12	3.67
**TDM**	7.77	9.59	8.44	9.26	9.49	8.17	6.29	7.38	8.87	9.86	8.86	8.92	9.87	9.63	8.74
**DFF**	83	111	109	83	105	103	89	87	102	108	101	101	95	93	98
**DPM**	114	137	137	116	131	131	120	117	131	135	133	131	127	124	127
**FLL**	29.8	31.9	28.7	26.5	34.8	27.0	30.8	28.3	33.0	28.4	24.6	27.5	31.5	25.8	29.2
**FLW**	1.37	1.35	1.40	1.52	1.51	1.34	1.34	1.30	1.47	1.34	1.32	1.43	1.33	1.28	1.38
**FLA**	30.5	32.3	30.0	30.1	39.2	27.0	31.1	27.6	36.3	28.6	24.3	29.6	31.4	24.6	30.2
**FLT**	0.297	0.347	0.318	0.329	0.303	0.309	0.338	0.298	0.318	0.309	0.329	0.280	0.283	0.310	0.312
**FLDW**	0.142	0.148	0.134	0.141	0.177	0.126	0.142	0.123	0.166	0.128	0.111	0.135	0.141	0.115	0.138
**SLA**	215.6	218.3	222.9	213.5	221.0	214.1	219.7	223.5	218.4	223.0	218.1	219.4	223.0	215.0	219.0
**SLW**	4.64	4.59	4.49	4.69	4.53	4.68	4.56	4.48	4.58	4.49	4.59	4.56	4.49	4.66	4.57
**CHLa**	2.03	2.28	2.17	2.34	2.23	1.99	1.81	2.05	2.15	2.21	2.15	2.15	2.26	2.32	2.15
**CHLb**	0.674	0.731	0.568	0.654	0.705	0.621	0.512	0.588	0.673	0.731	0.756	0.536	0.629	0.703	0.649
**TCHL**	2.70	3.01	2.74	2.99	2.93	2.61	2.33	2.64	2.83	2.94	2.91	2.69	2.89	3.03	2.80
**CAR**	0.568	0.661	0.644	0.701	0.648	0.569	0.554	0.636	0.601	0.624	0.589	0.676	0.710	0.656	0.631
**Pn**	18.1	19.9	18.9	19.7	19.1	18.1	18.8	17.9	20.0	19.8	19.3	19.6	19.4	21.3	19.3
**gs**	0.400	0.519	0.370	0.425	0.456	0.422	0.419	0.537	0.446	0.466	0.401	0.506	0.517	0.550	0.460
**Ci**	268.6	282.6	296.5	265.7	273.1	242.4	255.4	271.3	277.4	268.4	266.2	264.6	281.1	285.0	271.3
**E**	6.28	6.43	5.57	6.37	6.16	6.15	5.29	6.67	6.46	6.49	6.06	6.80	6.30	7.24	6.30
**FLN**	2.63	2.28	2.23	2.67	2.48	2.49	2.32	2.68	2.44	2.47	2.63	2.49	2.62	2.39	2.49
**PNUE**	14.9	19.3	19.3	15.9	17.4	15.8	17.9	15.0	18.2	18.1	16.2	17.5	16.6	19.4	17.3
**F_v_/F_m_**	0.799	0.812	0.792	0.805	0.804	0.794	0.799	0.803	0.799	0.804	0.803	0.802	0.801	0.814	0.802
**ϕPSII**	0.339	0.336	0.311	0.328	0.336	0.297	0.339	0.336	0.351	0.339	0.338	0.329	0.338	0.354	0.334
**ETR**	24.1	22.5	22.2	23.4	23.9	21.8	23.6	23.0	23.9	24.1	23.7	23.4	24.7	25.1	23.5
**qP**	0.546	0.519	0.527	0.514	0.541	0.474	0.527	0.537	0.564	0.563	0.544	0.545	0.555	0.563	0.537
**qN**	0.387	0.371	0.382	0.399	0.361	0.393	0.394	0.386	0.384	0.391	0.394	0.414	0.412	0.383	0.389
**GNU**	38.4	47.4	39.8	48.4	48.7	41.6	28.8	36.2	44.0	51.6	44.0	46.8	49.6	52.3	44.1
**SNU**	28.2	29.7	28.9	31.6	30.8	29.2	22.5	30.0	31.8	29.9	32.3	31.0	32.2	37.6	30.4
**TNU**	66.6	77.1	68.7	80.0	79.5	70.8	51.4	66.2	75.7	81.5	76.3	77.8	81.8	89.8	74.5
**AE**	12.7	23.4	15.4	11.4	21.1	20.5	11.6	13.2	6.8	27.3	7.5	23.3	29.2	13.9	17.0
**PE**	49.0	50.6	46.9	42.5	44.0	45.4	46.4	41.0	31.5	45.6	42.9	49.0	50.1	57.7	45.9
**PFP**	63.6	81.6	69.9	78.3	80.7	68.4	49.3	59.1	74.2	84.5	74.7	75.8	84.1	82.4	73.3
**ANRE**	29.3	49.3	37.2	30.7	51.5	45.2	26.4	31.9	21.1	61.4	20.2	49.8	60.3	28.1	38.7
**NUpE**	25.9	30.0	26.8	31.2	31.0	27.6	20.0	25.8	29.5	31.8	29.7	30.3	31.9	35.0	29.0
**NUtE**	47.9	53.4	51.1	49.0	51.3	48.4	48.2	44.9	49.0	52.2	49.1	49.2	51.6	46.0	49.4
**NUE_yield_**	12.4	15.9	13.6	15.3	15.7	13.3	9.6	11.5	14.5	16.5	14.6	14.8	16.4	16.1	14.3
**NUI**	116.8	125.4	123.2	115.8	120.3	115.7	123.1	111.9	117.3	121.9	116.6	115.7	121.4	107.5	118.0
**NHI**	57.7	61.6	58.2	60.5	61.3	58.8	56.0	54.7	58.0	63.0	57.6	60.0	60.8	58.2	59.0

Where, GY, Grain yield (t ha^-1^), TDM, Total dry matter (t ha^-1^); DFF, Days to 50% flowering; DPM, Days to physiological maturity; FLL, Flag leaf length (cm); FLW, Flag leaf width (cm); FLA, Flag leaf area (cm^2^); FLT, Flag leaf thickness (mm); FLDW, Flag leaf dry weight (g); SLA, Specific leaf area (cm^2^ g^-1^); SLW, Specific leaf weight (mg cm^-2^); CHLa, Chlorophyll a (mg g^-1^ fw); CHLb, Chlorophyll b (mg g^-1^ fw); TCHL,Total chlorophyll (mg g^-1^ fw); CAR, Carotenoids (mg g^-1^ fw); Pn, Photosynthetic rate (µmol [CO_2_] m^-2^ s^-1^); gs, Stomatal conductance (mol [H_2_O] m^-2^ s^-1^); E, Transpiration rate (mmol [H_2_O] m^-2^ s^-1^); Ci, Internal CO_2_ concentration (ppm); FLN, Flag leaf N content (%); PNUE, Photosynthetic nitrogen use efficiency (μmol [CO_2_] g^-1^ [N] s^-1^); F_v_/F_m_, Maximum quantum yield of PSII; ΦPSII, Actual quantum yield of PSII; ETR, Electron transport rate, qP-Coefficient of photochemical quenching; qN, Coefficient of non-photochemical quenching; GNU, Grain N uptake (kg N ha^-1^); SNU, Straw N uptake (kg N ha^-1^); TNU, Total plant N uptake (kg N ha^-1^); AE, Agronomic efficiency; PE, Physiological efficiency; PFP, Partial factor productivity; ANRE, Apparent nitrogen recovery efficiency; NUpE, Nitrogen uptake efficiency; NUtE, Nitrogen utilization efficiency; NUE_yield_, Nitrogen use efficiency_yield_; NUI, Nitrogen utilization index; NHI, Nitrogen harvest index.

**Table 6:** Cumulative mean values of morpho-physiological traits along with grain yield and NUE indices at N100 in different genotypes.

Genotype	Anjali	Birupa	Daya	Heera	Indira	Nidhi	N22	Tella Hamsa	V L Dhan 209	Vasumati	IR64	GQ25	Varadhan	MTU 1010	Mean
**GY**	4.80	4.82	5.31	5.13	5.73	4.86	3.28	3.74	5.49	5.45	5.10	5.08	5.66	5.84	5.02
**TDM**	10.00	10.51	11.11	10.99	11.86	10.34	7.37	8.36	11.73	11.65	10.61	10.90	12.08	11.55	10.65
**DFF**	84	112	111	85	107	104	91	88	103	110	102	103	97	94	99
**DPM**	114	138	138	118	133	133	122	119	133	137	135	132	128	125	129
**FLL**	34.8	34.5	30.7	30.7	37.2	29.4	34.2	32.7	35.7	32.2	27.8	31.1	35.7	27.9	32.5
**FLW**	1.43	1.53	1.44	1.60	1.54	1.48	1.44	1.37	1.58	1.46	1.36	1.55	1.41	1.35	1.47
**FLA**	37.4	39.5	33.0	36.8	43.0	32.6	37.1	33.5	42.2	35.4	28.3	36.2	37.9	28.4	35.8
**FLT**	0.320	0.370	0.351	0.350	0.334	0.339	0.371	0.338	0.345	0.346	0.359	0.323	0.312	0.340	0.342
**FLDW**	0.168	0.175	0.146	0.160	0.185	0.138	0.168	0.149	0.185	0.153	0.123	0.153	0.166	0.126	0.157
**SLA**	222.0	225.0	226.1	230.6	232.1	236.0	220.7	226.2	228.6	230.5	229.5	235.7	227.6	225.8	228.3
**SLW**	4.51	4.45	4.43	4.34	4.31	4.25	4.54	4.43	4.38	4.35	4.37	4.25	4.40	4.44	4.39
**CHLa**	2.36	2.67	2.55	2.68	2.75	2.43	2.32	2.52	2.64	2.78	2.77	2.67	3.03	2.82	2.64
**CHLb**	0.692	0.925	0.697	0.675	0.890	0.776	0.691	0.750	0.811	0.918	0.925	0.850	0.939	0.928	0.819
**TCHL**	3.05	3.60	3.24	3.35	3.64	3.20	3.01	3.27	3.45	3.70	3.69	3.52	3.97	3.75	3.46
**CAR**	0.695	0.722	0.734	0.834	0.774	0.681	0.673	0.714	0.743	0.766	0.731	0.736	0.833	0.782	0.744
**Pn**	23.5	23.6	23.4	23.8	24.1	22.1	22.0	23.3	23.8	23.7	23.8	24.0	24.5	26.0	23.7
**gs**	0.595	0.645	0.615	0.651	0.649	0.596	0.607	0.581	0.647	0.648	0.641	0.642	0.636	0.750	0.636
**Ci**	256.0	268.5	238.1	256.9	264.6	246.6	250.0	287.7	275.4	283.3	256.6	268.1	267.6	281.6	264.4
**E**	7.30	7.38	7.99	7.95	7.67	7.16	7.45	7.33	8.01	7.54	7.75	8.14	7.94	8.47	7.72
**FLN**	2.93	2.51	2.43	2.91	2.67	2.79	2.43	2.91	2.76	2.82	2.96	2.79	2.93	2.60	2.75
**PNUE**	18.0	21.4	22.3	19.0	21.9	18.9	20.1	18.2	19.9	19.5	18.6	20.3	19.2	22.6	20.0
**F_v_/F_m_**	0.813	0.820	0.814	0.818	0.811	0.819	0.805	0.817	0.815	0.812	0.811	0.817	0.817	0.823	0.815
**ϕPSII**	0.376	0.365	0.381	0.366	0.373	0.335	0.373	0.388	0.384	0.376	0.384	0.378	0.372	0.386	0.374
**ETR**	26.0	25.3	27.0	26.5	27.2	25.4	26.0	27.4	26.9	26.2	26.7	26.8	27.3	26.5	26.5
**qP**	0.588	0.585	0.608	0.580	0.579	0.553	0.593	0.596	0.617	0.615	0.616	0.618	0.608	0.622	0.598
**qN**	0.366	0.356	0.370	0.343	0.341	0.366	0.377	0.357	0.361	0.361	0.373	0.396	0.391	0.363	0.366
**GNU**	62.3	60.2	67.7	69.6	74.3	65.0	41.8	50.2	70.9	72.5	65.8	67.1	72.5	80.5	65.7
**SNU**	34.6	36.1	37.4	40.3	38.1	38.4	26.5	34.3	42.6	40.6	40.0	40.2	42.0	40.1	37.9
**TNU**	96.9	96.3	105.1	109.9	112.4	103.4	68.3	84.5	113.5	113.1	105.8	107.3	114.5	120.5	103.7
**AE**	22.6	19.1	25.9	17.9	27.6	24.7	14.0	14.4	21.3	25.9	17.4	24.5	29.1	24.2	22.0
**PE**	52.2	44.5	48.8	40.3	47.5	46.5	46.3	40.8	44.6	42.5	44.6	45.7	47.2	54.5	46.1
**PFP**	48.0	48.2	53.1	51.3	57.3	48.6	32.8	37.4	54.9	54.5	51.0	50.8	56.6	58.4	50.2
**ANRE**	44.9	43.9	55.0	45.3	58.6	55.2	30.1	34.2	48.4	62.3	39.5	54.3	62.9	44.8	48.5
**NUpE**	30.1	29.9	32.7	34.2	34.9	32.2	21.2	26.3	35.3	35.2	32.9	33.3	35.6	37.5	32.2
**NUtE**	49.8	50.2	50.8	46.8	51.4	47.4	48.0	44.1	48.6	48.6	48.5	47.7	49.7	48.5	48.6
**NUE_yield_**	14.9	15.0	16.5	16.0	17.8	15.1	10.2	11.6	17.1	16.9	15.9	15.8	17.6	18.2	15.6
**NUI**	104.0	109.5	106.2	100.5	106.4	101.0	107.8	98.8	103.8	104.2	101.1	102.4	106.1	96.0	103.4
**NHI**	64.3	62.5	64.5	63.3	66.0	62.7	61.1	59.3	62.4	64.1	62.0	62.4	63.4	66.7	63.2

Where, GY, Grain yield (t ha^-1^); TDM, Total dry matter (t ha^-1^); DFF, Days to 50% flowering; DPM, Days to physiological maturity; FLL, Flag leaf length (cm); FLW, Flag leaf width (cm); FLA, Flag leaf area (cm^2^); FLT, Flag leaf thickness (mm); FLDW, Flag leaf dry weight (g); SLA, Specific leaf area (cm^2^ g^-1^); SLW, Specific leaf weight (mg cm^-2^); CHLa, Chlorophyll a (mg g^-1^ fw); CHLb, Chlorophyll b (mg g^-1^ fw); TCHL, Total chlorophyll (mg g^-1^ fw); CAR, Carotenoids (mg g^-1^ fw); Pn, Photosynthetic rate (µmol [CO_2_] m^-2^ s^-1^); gs, Stomatal conductance (mol [H_2_O] m^-2^ s^-1^); E, Transpiration rate (mmol [H_2_O] m^-2^ s^-1^); Ci, Internal CO_2_ concentration (ppm); FLN, Flag leaf N content (%); PNUE, Photosynthetic nitrogen use efficiency (μmol [CO_2_] g^-1^ [N] s^-1^); F_v_/F_m_, Maximum quantum yield of PSII; ΦPSII, Actual quantum yield of PSII; ETR, Electron transport rate; qP, Coefficient of photochemical quenching; qN, Coefficient of non-photochemical quenching; GNU, Grain N uptake (kg N ha^-1^); SNU, Straw N uptake (kg N ha^-1^); TNU, Total plant N uptake (kg N ha^-1^); AE, Agronomic efficiency; PE, Physiological efficiency; PFP, Partial factor productivity; ANRE, Apparent nitrogen recovery efficiency; NUpE, Nitrogen uptake efficiency; NUtE, Nitrogen utilization efficiency; NUE_yield_, Nitrogen use efficiency_yield_; NUI, Nitrogen utilization index; NHI, Nitrogen harvest index.

**Table 7:** Cumulative mean values of morpho-physiological traits along with grain yield and NUE indices at N150 in different genotypes.

Genotype	Anjali	Birupa	Daya	Heera	Indira	Nidhi	N22	Tella Hamsa	V L Dhan 209	Vasumati	IR64	GQ25	Varadhan	MTU 1010	Mean
**GY**	4.91	5.16	5.24	5.00	5.83	4.64	3.48	3.88	5.30	5.66	5.47	5.12	5.72	5.81	5.09
**TDM**	10.56	11.26	11.08	10.86	11.98	10.32	7.89	8.75	11.39	12.02	11.23	11.15	12.48	11.43	10.89
**DFF**	86	113	112	86	108	105	92	89	105	111	104	105	98	95	101
**DPM**	116	140	140	119	134	134	123	120	134	138	135	134	129	127	130
**FLL**	39.3	35.1	32.3	34.7	39.5	31.6	37.3	34.3	39.0	35.2	30.8	33.6	38.9	31.5	35.2
**FLW**	1.54	1.60	1.50	1.61	1.72	1.59	1.48	1.43	1.67	1.52	1.46	1.70	1.45	1.44	1.55
**FLA**	45.4	42.1	36.2	41.9	50.9	37.7	41.4	36.8	48.8	40.2	33.8	42.9	42.5	34.2	41.1
**FLT**	0.348	0.420	0.377	0.393	0.377	0.354	0.382	0.352	0.393	0.381	0.400	0.338	0.351	0.374	0.374
**FLDW**	0.190	0.179	0.152	0.173	0.211	0.151	0.172	0.157	0.202	0.167	0.139	0.172	0.171	0.147	0.170
**SLA**	239.3	235.7	237.6	241.7	241.7	248.8	240.1	235.3	242.0	240.1	243.3	248.7	248.9	232.5	241.1
**SLW**	4.19	4.25	4.21	4.14	4.14	4.03	4.17	4.25	4.13	4.17	4.12	4.03	4.02	4.30	4.15
**CHLa**	2.86	3.01	2.74	2.84	2.99	2.74	2.66	2.68	3.05	3.06	3.04	2.91	2.99	3.13	2.91
**CHLb**	0.921	0.943	0.855	0.921	1.019	0.945	0.768	0.890	0.951	0.945	0.954	0.912	0.930	0.943	0.921
**TCHL**	3.78	3.95	3.60	3.76	4.01	3.68	3.43	3.57	4.00	4.00	3.99	3.82	3.92	4.07	3.83
**CAR**	0.785	0.821	0.730	0.791	0.813	0.729	0.774	0.757	0.846	0.857	0.808	0.792	0.831	0.893	0.802
**Pn**	25.8	25.1	24.4	25.6	26.1	23.2	23.8	25.5	25.6	25.2	25.1	25.8	26.6	27.3	25.4
**gs**	0.655	0.667	0.693	0.741	0.680	0.625	0.671	0.710	0.679	0.720	0.717	0.771	0.790	0.767	0.706
**E**	8.65	8.53	8.21	8.39	8.29	7.44	7.62	8.57	8.56	8.92	8.80	8.92	8.04	8.95	8.42
**Ci**	247.5	251.5	242.1	257.4	246.2	253.3	228.7	264.8	269.4	256.8	268.8	269.0	273.7	273.2	257.3
**FLN**	3.03	2.67	2.63	3.03	2.82	2.92	2.54	2.96	2.81	2.91	3.07	2.92	3.02	2.75	2.86
**PNUE**	20.7	22.4	22.6	20.6	22.9	19.9	22.8	20.4	22.3	21.0	20.0	22.0	22.2	23.2	21.6
**F_v_/F_m_**	0.822	0.821	0.821	0.819	0.819	0.821	0.813	0.823	0.819	0.819	0.814	0.820	0.821	0.832	0.820
**ϕPSII**	0.394	0.376	0.404	0.386	0.405	0.363	0.380	0.392	0.409	0.384	0.395	0.390	0.380	0.401	0.390
**ETR**	26.7	26.1	28.1	28.2	29.0	26.3	26.6	28.4	28.3	26.7	27.7	27.4	28.4	27.4	27.5
**qP**	0.617	0.609	0.649	0.615	0.607	0.557	0.629	0.636	0.634	0.643	0.649	0.639	0.648	0.655	0.628
**qN**	0.362	0.334	0.356	0.328	0.329	0.349	0.352	0.351	0.344	0.349	0.358	0.368	0.362	0.333	0.348
**GNU**	66.5	67.5	69.4	70.3	78.0	63.3	45.9	54.3	69.3	79.1	72.7	69.8	78.0	83.4	69.1
**SNU**	40.0	42.1	40.6	42.2	42.0	40.8	29.6	37.8	42.6	43.4	42.7	42.5	47.1	43.2	41.2
**TNU**	106.5	109.6	110.0	112.5	120.0	104.1	75.6	92.1	111.9	122.5	115.4	112.3	125.1	126.6	110.3
**AE**	15.8	15.0	16.8	11.1	19.0	15.0	10.6	10.6	12.9	18.7	14.1	16.6	19.8	15.9	15.1
**PE**	43.8	40.9	42.9	35.1	43.9	40.6	42.5	37.0	40.6	39.6	43.2	42.4	41.8	47.1	41.5
**PFP**	32.7	34.4	35.0	33.4	38.9	30.9	23.2	25.9	35.3	37.7	36.5	34.1	38.1	38.8	33.9
**ANRE**	36.4	38.1	39.9	31.9	44.2	37.3	24.9	27.9	31.2	47.8	32.8	39.6	49.0	33.9	36.8
**NUpE**	28.0	28.8	28.9	29.6	31.6	27.4	19.9	24.2	29.4	32.2	30.4	29.5	32.9	33.3	29.0
**NUtE**	46.1	47.5	47.8	44.7	49.0	44.7	46.0	42.2	47.4	46.5	47.6	45.8	46.1	45.9	46.2
**NUE_yield_**	12.9	13.6	13.8	13.2	15.3	12.2	9.1	10.2	13.9	14.9	14.4	13.5	15.1	15.3	13.4
**NUI**	99.1	103.7	101.2	97.0	100.8	99.6	104.3	95.0	102.1	99.1	98.3	99.7	100.5	90.3	99.3
**NHI**	62.5	61.6	63.0	62.6	65.0	60.7	60.8	59.0	61.8	64.6	62.7	62.1	62.5	65.8	62.5

Where, GY, Grain yield (t ha^-1^); TDM, Total dry matter (t ha^-1^); DFF, Days to 50% flowering; DPM, Days to physiological maturity; FLL, Flag leaf length (cm); FLW, Flag leaf width (cm); FLA, Flag leaf area (cm^2^); FLT, Flag leaf thickness (mm); FLDW, Flag leaf dry weight (g); SLA, Specific leaf area (cm^2^ g^-1^); SLW, Specific leaf weight (mg cm^-2^); CHLa, Chlorophyll a (mg g^-1^ fw); CHLb, Chlorophyll b (mg g^-1^ fw); TCHL, Total chlorophyll (mg g^-1^ fw); CAR, Carotenoids (mg g^-1^ fw); Pn, Photosynthetic rate (µmol [CO_2_] m^-2^ s^-1^); gs, Stomatal conductance (mol [H_2_O] m^-2^ s^-1^); E, Transpiration rate (mmol [H_2_O] m^-2^ s^-1^); Ci, Internal CO_2_ concentration (ppm); FLN, Flag leaf N content (%); PNUE, Photosynthetic nitrogen use efficiency (μmol [CO_2_] g^-1^ [N] s^-1^); F_v_/F_m_, Maximum quantum yield of PSII; ΦPSII, Actual quantum yield of PSII; ETR, Electron transport rate; qP, Coefficient of photochemical quenching; qN, Coefficient of non-photochemical quenching; GNU, Grain N uptake (kg N ha^-1^); SNU, Straw N uptake (kg N ha^-1^); TNU, Total plant N uptake (kg N ha^-1^); AE, Agronomic efficiency; PE, Physiological efficiency; PFP, Partial factor productivity; ANRE, Apparent nitrogen recovery efficiency; NUpE, Nitrogen uptake efficiency; NUtE, Nitrogen utilization efficiency; NUE_yield_, Nitrogen use efficiency_yield_; NUI, Nitrogen utilization index; NHI, Nitrogen harvest index.

**Table 8:** Range and mean values of morpho-physiological traits along with grain yield and NUE indices at graded N application for cumulative data of four seasons.

Trait	N0			N50			N100			N150		
	Min	Max	Mean	Min	Max	Mean	Min	Max	Mean	Min	Max	Mean
**Grain yield (g m^-2^)**	1.88	3.43	2.82	2.46	4.22	3.67	3.28	5.84	5.02	3.48	5.83	5.09
**Total dry matter (g m^-2^)**	5.29	9.18	7.43	6.29	9.87	8.74	7.37	12.08	10.65	7.89	12.48	10.89
**Days to 50% flowering**	81	109	96	83	111	98	84	112	99	86	113	101
**Days to maturity**	112	136	125	114	137	127	114	138	129	116	140	130
**Flag leaf length (cm)**	23.7	31.0	26.3	24.6	34.8	29.2	27.8	37.2	32.5	30.8	39.5	35.2
**Flag leaf width (cm)**	1.16	1.44	1.28	1.28	1.52	1.38	1.35	1.60	1.47	1.43	1.72	1.55
**Flag leaf area (cm^2^)**	20.8	31.6	25.4	24.3	39.2	30.2	28.3	43.0	35.8	33.8	50.9	41.1
**Flag leaf thickness (mm)**	0.238	0.329	0.281	0.280	0.347	0.312	0.312	0.371	0.342	0.338	0.420	0.374
**Flag leaf dry weight (g)**	0.098	0.154	0.121	0.111	0.177	0.138	0.123	0.185	0.157	0.139	0.211	0.170
**Specific leaf area (cm^2^ g^-1^)**	198.2	215.3	209.0	213.5	223.5	219.0	220.7	236.0	228.3	232.5	248.9	241.1
**Specific leaf weight (mg cm^-2^)**	4.66	5.05	4.80	4.48	4.69	4.57	4.25	4.54	4.39	4.02	4.30	4.15
**Chlorophyll a content (mg g^-1^ fw)**	1.53	1.89	1.71	1.81	2.34	2.15	2.32	3.03	2.64	2.66	3.13	2.91
**Chlorophyll b content (mg g^-1^ fw)**	0.370	0.552	0.478	0.512	0.756	0.649	0.675	0.939	0.819	0.768	1.019	0.921
**Total Chlorophyll content (mg g^-1^ fw)**	1.96	2.41	2.19	2.33	3.03	2.80	3.01	3.97	3.46	3.43	4.07	3.83
**Carotenoid content (mg g^-1^ fw)**	0.457	0.582	0.510	0.554	0.710	0.631	0.673	0.834	0.744	0.729	0.893	0.802
**Photosynthetic rate (µmol [CO_2_] m^-2^ s^-1^)**	14.4	16.7	15.7	17.9	21.3	19.3	22.0	26.0	23.7	23.2	27.3	25.4
**Stomatal conductance (mol [H_2_O] m^-2^ s^-1^)**	0.238	0.322	0.281	0.370	0.550	0.460	0.581	0.750	0.636	0.625	0.790	0.706
**Transpiration rate (mmol [H_2_O] m^-2^ s^-1^)**	4.15	5.50	4.94	5.29	7.24	6.30	7.16	8.47	7.72	7.44	8.95	8.42
**Internal CO_2_ concentration (ppm)**	263.1	306.4	281.1	242.4	296.5	271.3	238.1	287.7	264.4	228.7	273.7	257.3
**Flag leaf N (%) content**	1.92	2.35	2.11	2.23	2.68	2.49	2.43	2.96	2.75	2.54	3.07	2.86
**Photosynthetic nitrogen use efficiency (μmol [CO_2_] g^-1^ [N] s^-1^)**	13.3	17.6	15.8	14.9	19.4	17.3	18.0	22.6	20.0	19.9	23.2	21.6
**Maximum quantum yield of PSII**	0.780	0.807	0.792	0.792	0.814	0.802	0.805	0.823	0.815	0.813	0.832	0.820
**Actual quantum yield of PSII**	0.288	0.338	0.309	0.297	0.354	0.334	0.335	0.388	0.374	0.363	0.409	0.390
**Electron transport rate**	20.3	23.2	21.5	21.8	25.1	23.5	25.3	27.4	26.5	26.1	29.0	27.5
**Coefficient of photochemical quenching**	0.474	0.517	0.499	0.474	0.564	0.537	0.553	0.622	0.598	0.557	0.655	0.628
**Coefficient of non-photochemical quenching**	0.391	0.464	0.426	0.361	0.414	0.389	0.341	0.396	0.366	0.328	0.368	0.348
**Grain N uptake (kg N ha^-1^)**	20.3	41.4	30.6	28.8	52.3	44.1	41.8	80.5	65.7	45.9	83.4	69.1
**Straw N uptake (kg N ha^-1^)**	17.9	34.3	24.5	22.5	37.6	30.4	26.5	42.6	37.9	29.6	47.1	41.2
**Total plant N uptake (kg N ha^-1^)**	38.2	75.7	55.1	51.4	89.8	74.5	68.3	120.5	103.7	75.6	126.6	110.3
**Agronomic efficiency**	-	–	-	6.8	29.2	17.0	14.0	29.1	22.0	10.6	19.8	15.1
**Physiological efficiency**	-	–	-	31.5	57.7	45.9	40.3	54.5	46.1	35.1	47.1	41.5
**Partial factor productivity**	-	–	-	49.3	84.5	73.3	32.8	58.4	50.2	23.2	38.9	33.9
**Apparent nitrogen recovery efficiency**	-	–	-	20.2	61.4	38.7	30.1	62.9	48.5	24.9	49.0	36.8
**Nitrogen uptake efficiency**	19.9	39.5	28.8	20.0	35.0	29.0	21.2	37.5	32.2	19.9	33.3	29.0
**Nitrogen utilization efficiency**	45.3	56.4	51.5	44.9	53.4	49.4	44.1	51.4	48.6	42.2	49.0	46.2
**Nitrogen use efficiency**	9.8	17.9	14.7	9.6	16.5	14.3	10.2	18.2	15.6	9.1	15.3	13.4
**Nitrogen utilization index**	121.3	148.3	136.1	107.5	125.4	118.0	96.0	109.5	103.4	90.3	104.3	99.3
**Nitrogen harvest index**	49.0	60.6	55.4	54.7	63.0	59.0	59.3	66.7	63.2	59.0	65.8	62.5

Flag leaf length, width, area, thickness, and dry weight increased significantly with increased application of N. From N0 to N150, mean values of flag leaf length increased from 26.3 to 35.2 cm, flag leaf width increased from 1.28 to 1.55 cm, flag leaf area increased from 25.4 to 41.1 cm^2^, flag leaf thickness increased from 0.281 to 0.374 mm, and flag leaf dry weight increased from 0.121 to 0.170 g. Among the genotypes, Indira exhibited the highest flag leaf length (34.8 and 37.2 cm), area (39.2 and 43.0 cm^2^), and dry weight (0.177 and 0.185 g), while the lowest flag leaf length (24.6 and 27.8 cm), area (24.3 and 28.3 cm^2^), and dry weight (0.111 and 0.123 g) were observed in IR64, at N50 and N100. The highest flag leaf width (1.52 and 1.60 cm) was noticed in Heera at N50 and N100, whereas MTU 1010 exhibited the lowest values (1.28 and 1.35 cm). Flag leaf thickness was the highest (0.347 mm) in Birupa and the lowest in GQ25 (0.280 mm) at N50, and the highest (0.371 mm) in N22 and the lowest (0.312 mm) in Varadhan at N100. SLA increased significantly with increased application of N whereas significant reduction in SLW is observed. From N0 to N150, mean values of SLA increased from 209.0 to 241.1 cm^2^ g^−1^ and SLW decreased from 4.80 to 4.15 mg cm^−2^. Among the genotypes, SLA ranged from 213.5 (Heera) to 223.5 cm^2^ g^−1^ (Tella Hamsa) at N50, and from 220.7 (N22) to 236.0 cm^2^ g^−1^ (Nidhi) at N100. SLW ranged from 4.48 (Tella Hamsa) to 4.69 mg cm^−2^ (Heera) at N50, and from 4.25 (Nidhi) to 4.54 mg cm^−2^ (N22) at N100.

#### Photosynthetic pigment content

Among the treatments, mean contents of chlorophyll a increased significantly from 1.71 to 2.91 mg g^−1^ fw, chlorophyll b increased significantly from 0.478 to 0.921 mg g^−1^ fw, total chlorophyll increased significantly from 2.19 to 3.83 mg g^−1^ fw, and carotenoid increased significantly from 0.510 to 0.802 mg g^−1^ fw with the increase in N application from N0 to N150. Among the genotypes, the highest chlorophyll a content (2.34 and 3.03 mg g^−1^ fw) was recorded in Heera at N50 and Varadhan at N100 while the lowest content (1.81 and 2.32 mg g^−1^ fw) was recorded in N22 at N50 and N100. IR64 at N50 and Varadhan at N100 recorded the highest contents of chlorophyll b (0.756 and 0.939 mg g^−1^ fw),whereas N22 at N50 and Heera at N100 recorded the lowest contents (0.512 and 0.675 mg g^−1^ fw). Total chlorophyll content was the lowest (2.33 and 3.01 mg g^−1^ fw) in N22 at N50 and N100 and the highest (3.03 and 3.97 mg g^−1^ fw) in MTU 1010 at N50 and Varadhan at N100. Carotenoid content was the highest (0.710 and 0.834 mg g^−1^ fw) in Varadhan at N50 and Heera at N100 and the lowest (0.554 and 0.673 mg g^−1^ fw) in N22 at N50 and N100.

#### Flag leaf N content and gas exchange traits

Mean Pn increased significantly from 15.7 to 25.4 µmol (CO_2_) m^−2^ s^−1^, g_s_ increased significantly from 0.281 to 0.706 mol (H_2_O) m^−2^ s^−1^, and E increased significantly from 4.94 to 8.42 mmol (H_2_O) m^−2^ s^−1^ while C_i_ decreased significantly from 279.6 to 256.5 ppm with an increase in N application from N0 to N150. MTU 1010 exhibited the highest Pn [1.3 and 26.0 µmol (CO_2_) m^−2^ s^−1^], g_s_ [0.550 and 0.750 mol (H_2_O) m^−2^ s^−1^], and E [7.24 and 8.47 mmol (H_2_O) m^−2^ s^−1^], and the highest C_i_ (296.5 and 287.7 ppm) was observed in Daya at N50 and Tella Hamsa at N100, while the lowest Pn [17.9 and 22.0 µmol (CO_2_) m^−2^ s^−1^] was observed in Tella Hamsa at N50 and N22 at N100, the lowest g_s_ [0.370 and 0.581 mol (H_2_O) m^−2^ s^−1^] was recorded in Daya at N50 and Tella Hamsa at N100, the lowest E [5.29 and 7.16 mmol (H_2_O) m^−2^ s^−1^] was noticed in N22 at N50 and Nidhi at N100, and the lowest C_i_ (242.4 and 238.1 ppm) was recorded in Nidhi at N50 and Daya at N100. Among the treatments, mean flag leaf N content increased significantly from 2.11% to 2.86% and mean PNUE increased significantly from 15.8 to 21.6 μmol (CO_2_) g^−1^ N s^−1^ with an increase in N application from N0 to N150. Flag leaf N content was the highest (2.68% and 2.96%) in Tella Hamsa at N50 and IR64 at N100 and the lowest (2.23% and 2.43%) in Daya at N50 and N100. MTU 1010 exhibited the highest PNUE [19.4 and 22.6 μmol (CO_2_) g^−1^ N s^−1^] at N50 and N100 whereas Anjali exhibited the lowest [14.9 and 18.0 μmol (CO_2_) g^−1^ N s^−1^].

#### Chlorophyll fluorescence traits

F_v_/F_m_, ΦPSII, ETR, and qP have increased significantly with an increase in application of N, whereas qN has significantly decreased. Mean values of F_v_/F_m_ increased from 0.792 to 0.820, ΦPSII increased from 0.309 to 0.390, ETR increased from 21.5 to 27.5, qP increased from 0.499 to 0.628, and qN decreased from 0.426 to 0.348 with increased N application from N0 to N150. Among the genotypes, MTU 1010 recorded the highest F_v_/F_m_ (0.814 and 0.823) at N50 and N100, whereas Daya at N50 and N22 at N100 recorded the lowest (0.792 and 0.805). ΦPSII was the highest (0.354 and 0.388) in MTU 1010 at N50 and Tella Hamsa at N100 and the lowest (0.297 and 0.335) in Nidhi at N50 and N100. MTU 1010 at N50 and Tella Hamsa at N100 recorded the highest ETR (25.1 and 27.4) whereas Nidhi at N50 and Birupa at N100 recorded the lowest (21.8 and 25.3). qP was the highest (0.564 and 0.622) in VL Dhan 209 at N50 and MTU 1010 at N100 and the lowest (0.474 and 0.553) in Nidhi at N50 and N100. GQ25 exhibited the highest qN (0.414 and 0.396) at N50 and N100 whereas Indira exhibited the lowest qN (0.361 and 0.341).

#### Nitrogen uptake and NUE indices

Increased N application from N0 to N150 resulted in a significant increase in mean grain N uptake from 30.6 to 69.1 kg N ha^−1^, mean straw N uptake from 24.5 to 41.2 kg N ha^−1^, and as total plant from 55.1 to 110.3 kg N ha^−1^. Grain N uptake ranged from 28.8 (N22) to 52.3 kg N ha^−1^ (MTU 1010) at N50, and from 41.8 (N22) to 80.5 kg N ha^−1^ (MTU 1010) at N100. Straw N uptake ranged from 22.5 (N22) to 37.6 kg N ha^−1^ (MTU 1010) at N50, and from 26.5 (N22) to 42.6 kg N ha^−1^ (VL Dhan 209) at N100. Total N uptake ranged from 51.4 (N22) to 89.8 kg N ha^−1^ (MTU 1010) at N50, and from 68.3 (N22) to 120.5 kg N ha^−1^ (MTU 1010) at N100.

#### Multiple correlation analysis

**Figure 1:**
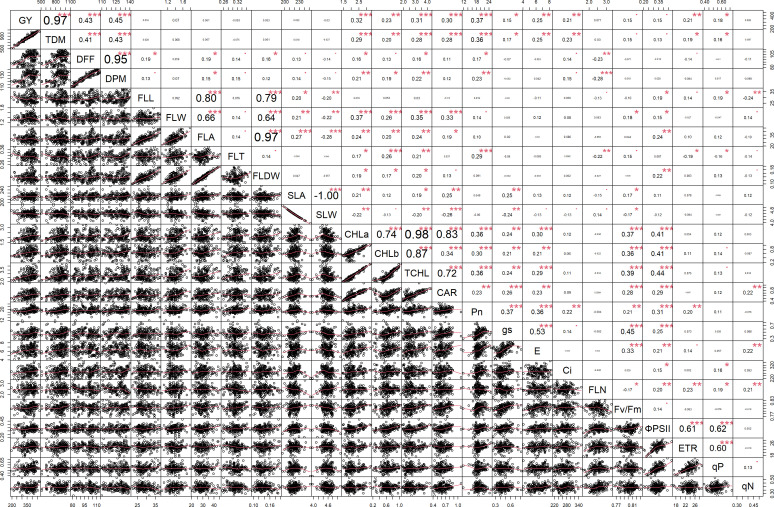
Correlation among the morpho-physiological parameters along with grain yield at N50. GY, Grain yield; TDM, Total dry matter; DFF, Days to 50% flowering; DPM, Days to physiological maturity; FLL, Flag leaf length; FLW, Flag leaf width; FLA, Flag leaf area; FLT, Flag leaf thickness; FLDW, Flag leaf dry weight; SLA, Specific leaf area; SLW, Specific leaf weight; CHLa, Chlorophyll a; CHLb, Chlorophyll b; TCHL, Total chlorophyll; CAR, Carotenoids; Pn, Photosynthetic rate; g_s_, Stomatal conductance; E, Transpiration rate; C_i_, Internal CO_2_ concentration; FLN, Flag leaf N content; PNUE, Photosynthetic nitrogen use efficiency; F_v_/F_m_, Maximum quantum yield of PSII; ΦPSII, Actual quantum yield of PSII; ETR, Electron transport rate; qP, Coefficient of photochemical quenching; qN, Coefficient of non-photochemical quenching. *** - p≤ 0.001, **- p≤ 0.01, *- p≤ 0.05, • - p≤ 0.1.

**Figure 2:**
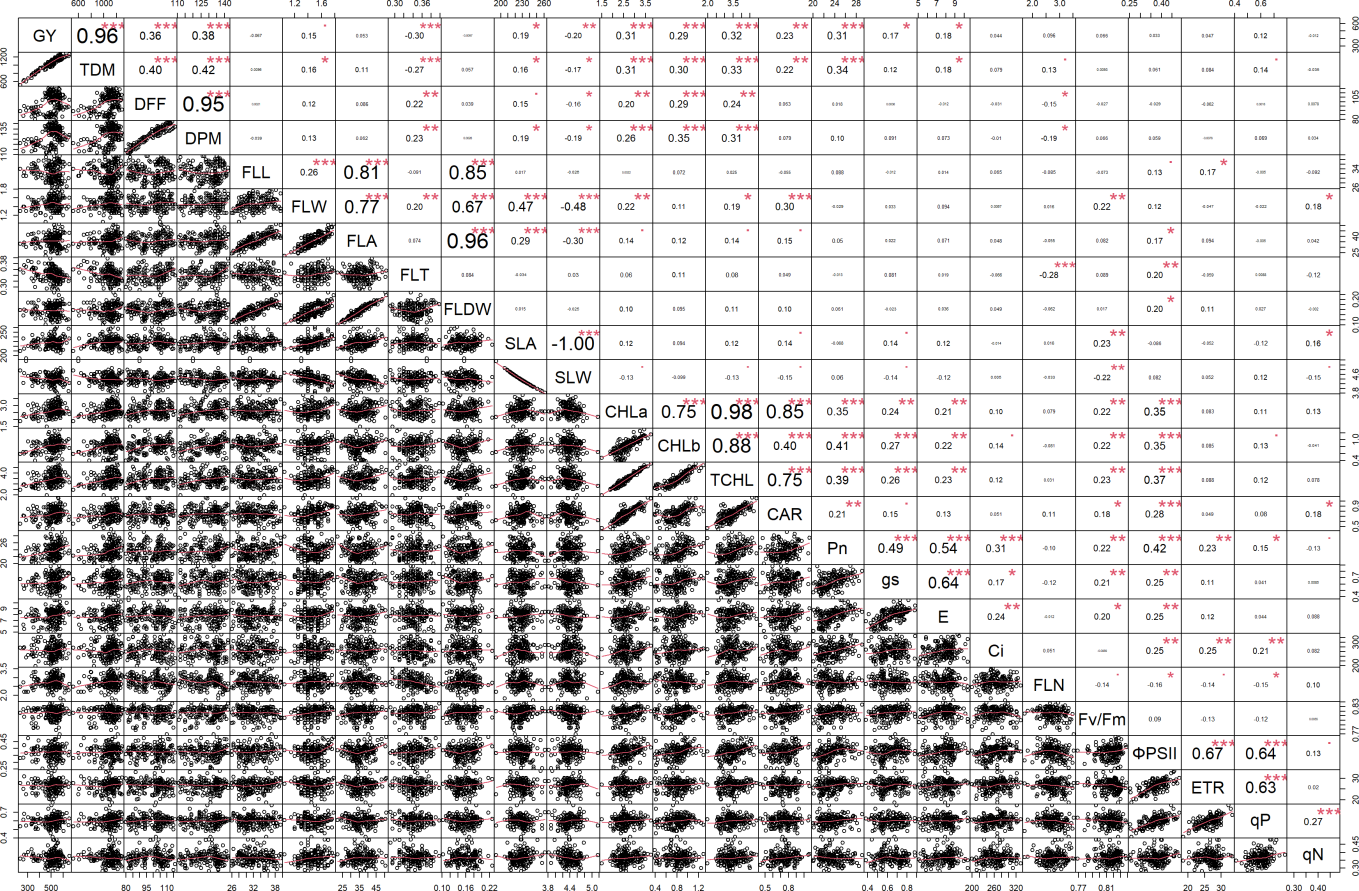
Correlation among the morpho-physiological parameters along with grain yield at N100. GY, Grain yield; TDM, Total dry matter; DFF, Days to 50% flowering; DPM, Days to physiological maturity; FLL, Flag leaf length; FLW, Flag leaf width; FLA, Flag leaf area; FLT, Flag leaf thickness; FLDW, Flag leaf dry weight; SLA, Specific leaf area; SLW, Specific leaf weight; CHLa, Chlorophyll a; CHLb, Chlorophyll b; TCHL, Total chlorophyll; CAR, Carotenoids; Pn, Photosynthetic rate; g_s_, Stomatal conductance; E, Transpiration rate; C_i_, Internal CO_2_ concentration; FLN, Flag leaf N content; PNUE, Photosynthetic nitrogen use efficiency; F_v_/F_m_, Maximum quantum yield of PSII; ΦPSII, Actual quantum yield of PSII; ETR, Electron transport rate; qP, Coefficient of photochemical quenching; qN, Coefficient of non-photochemical quenching. *** - p≤ 0.001, **- p≤ 0.01, *- p≤ 0.05, • - p≤ 0.1.

Multiple correlation analysis (**Figures 1**, **2**) of morpho-physiological traits along with grain yield separately at N50 and N100 indicates that several traits were highly significantly correlated with grain yield in both N treatments. Interestingly, the correlations of F_v_/F_m_, ΦPSII, ETR, qP, and C_i_ with grain yield were only significant at N50. Furthermore, ΦPSII, ETR, and qP showed a significant negative correlation and qN noted a non-significant positive correlation with flag leaf nitrogen (FLN) at N100. In contrast, ΦPSII, ETR, qP, and qN noted a significant positive correlation with FLN at N50.

#### Correlation of grain yield with NUE indices

**Figure 3:**
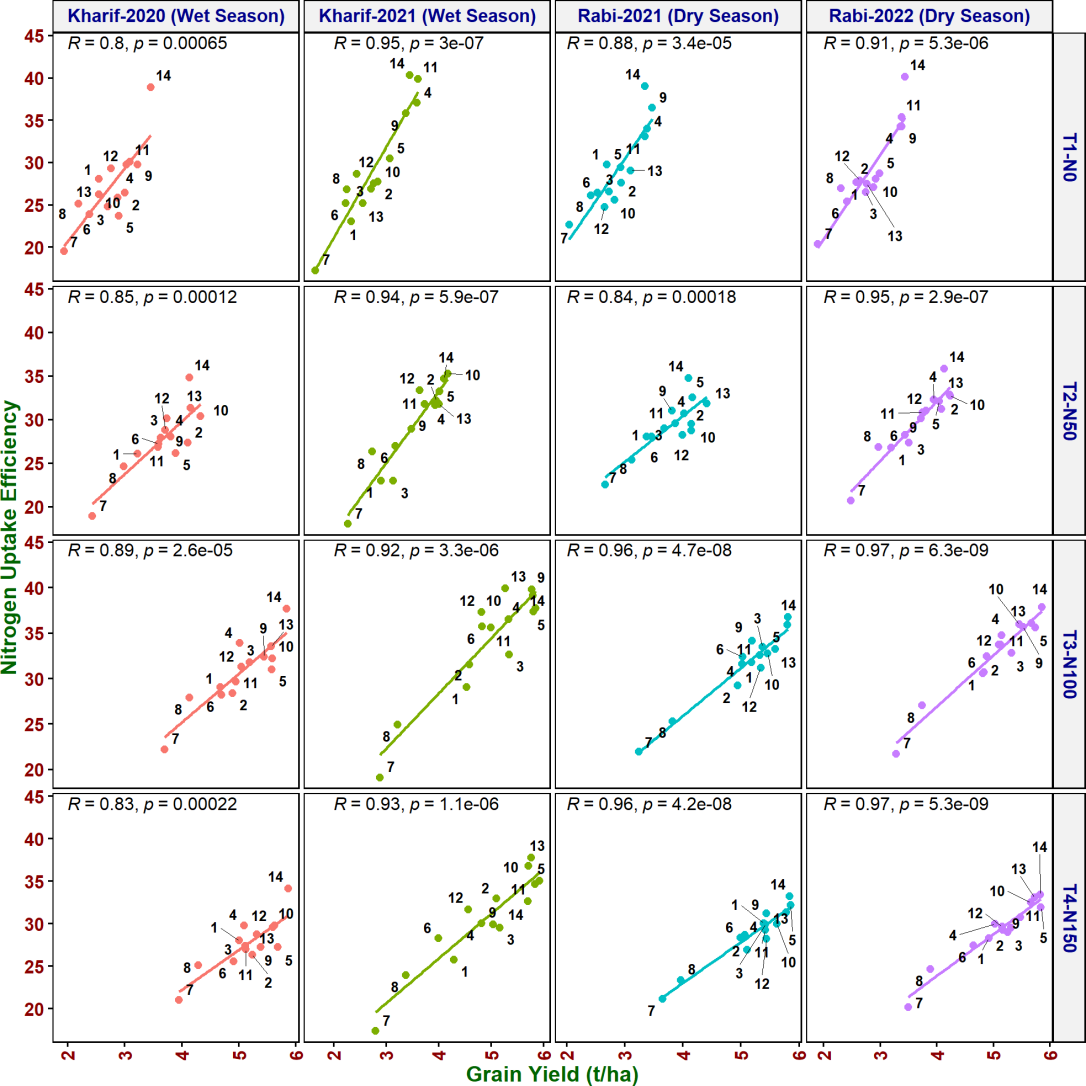
Relationship between nitrogen uptake efficiency (NUpE) and grain yield of rice genotypes at different N levels and seasons. 1, Anjali; 2, Birupa; 3, Daya; 4, Heera; 5, Indira; 6, Nidhi; 7, N22; 8, Tella Hamsa; 9, VL Dhan 209; 10, Vasumati; 11, IR64; 12, GQ25; 13, Varadhan; and 14, MTU 1010.

**Figure 4:**
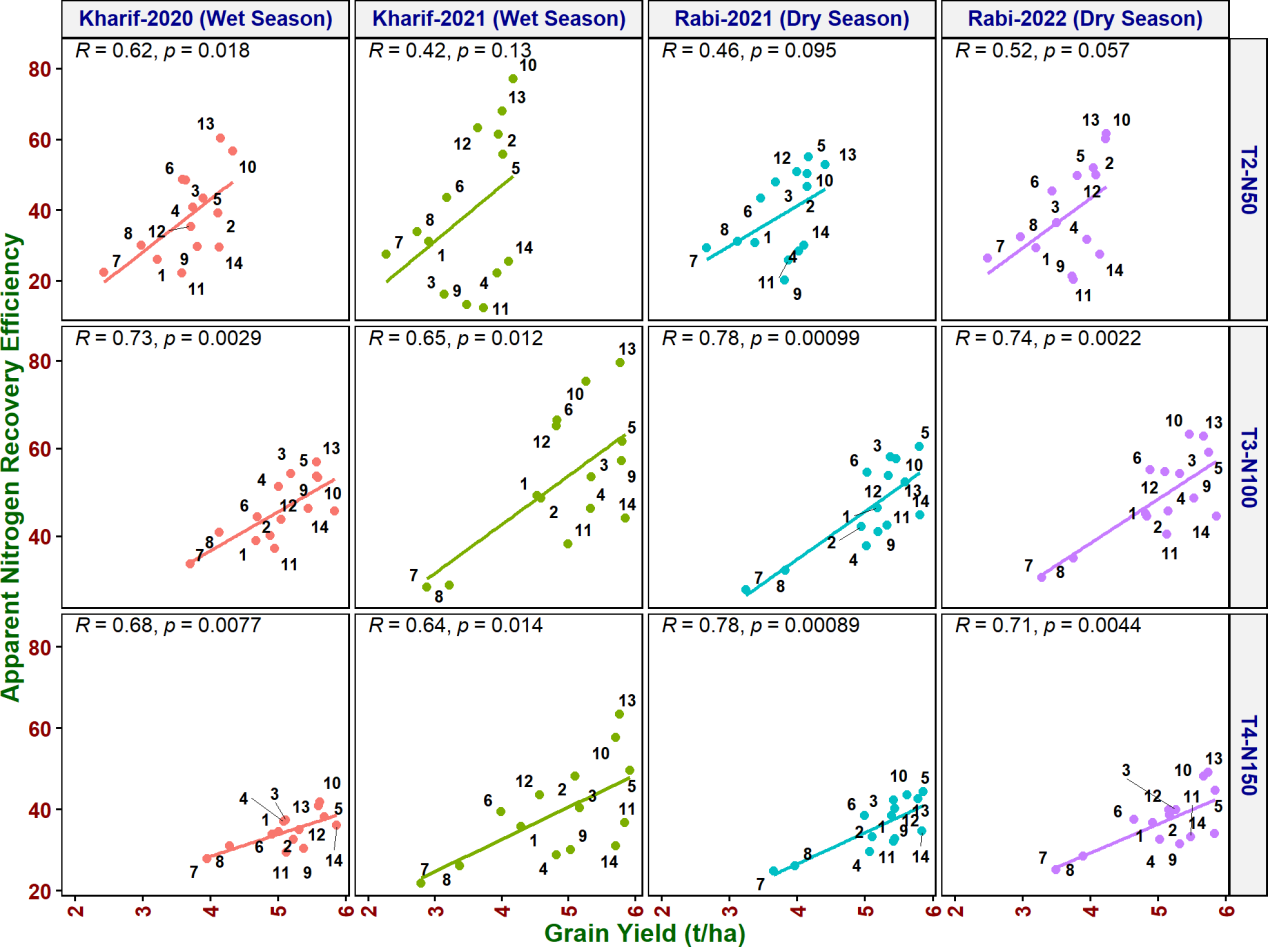
Relationship between apparent nitrogen recovery efficiency (ANRE) and grain yield of rice genotypes at different N levels and seasons. 1, Anjali; 2, Birupa; 3, Daya; 4, Heera; 5, Indira; 6, Nidhi; 7, N22; 8, Tella Hamsa; 9, VL Dhan 209; 10, Vasumati; 11, IR64; 12, GQ25; 13, Varadhan; and 14, MTU 1010.

**Figure 5:**
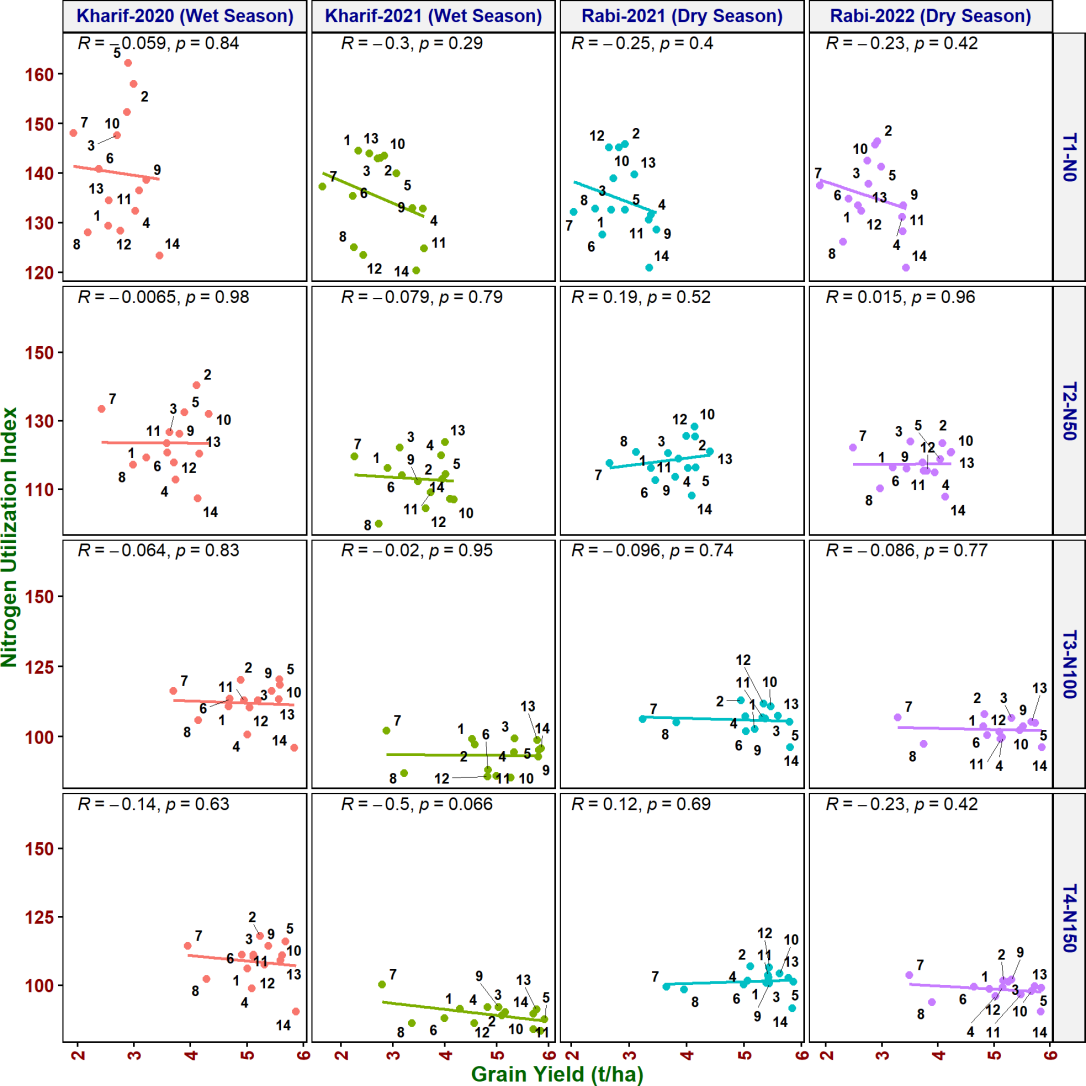
Relationship between nitrogen utilization index (NUI) and grain yield of rice genotypes at different N levels and seasons. 1, Anjali; 2, Birupa; 3, Daya; 4, Heera; 5, Indira; 6, Nidhi; 7, N22; 8, Tella Hamsa; 9, VL Dhan 209; 10, Vasumati; 11, IR64; 12, GQ25; 13, Varadhan; and 14, MTU 1010.

**Figure 6:**
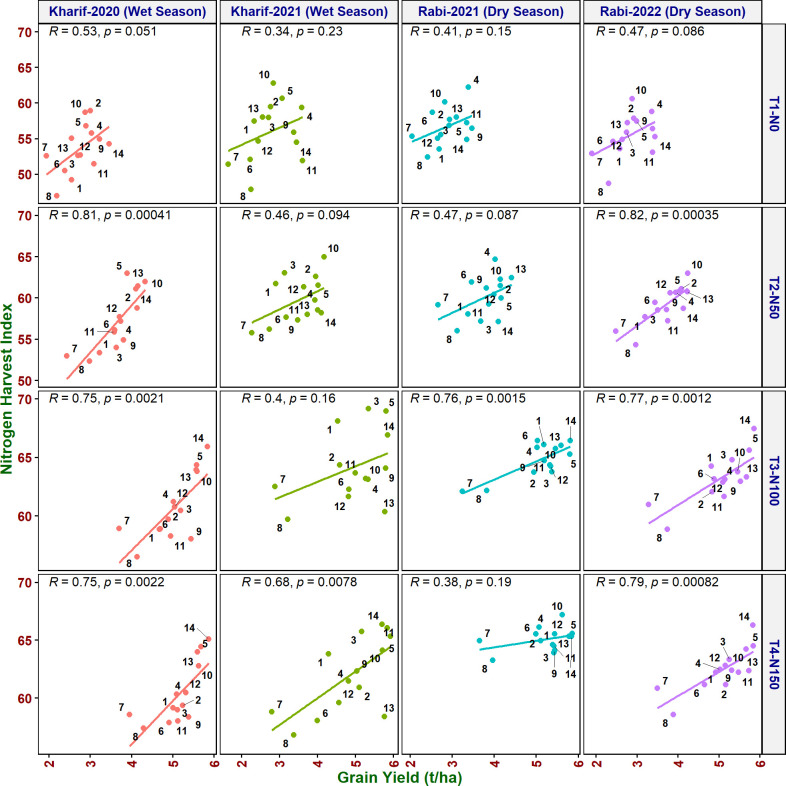
Relationship between nitrogen harvest index (NHI) and grain yield of rice genotypes at different N levels and seasons. 1, Anjali; 2, Birupa; 3, Daya; 4, Heera; 5, Indira; 6, Nidhi; 7, N22; 8, Tella Hamsa; 9, VL Dhan 209; 10, Vasumati; 11, IR64; 12, GQ25; 13, Varadhan; and 14, MTU 1010.

As it is inevitable to reduce N fertilizer application by 50% in agriculture for environmental sustainability, in addition to the above traits, various NUE indices were also calculated to identify their applicability to assess the genotypes. Grain yield value is not required to derive NUpE, ANRE, NUI, and NHI. Therefore, these four indices along with other indices were compared with grain yield to assess their suitability to identify promising genotypes at reduced N cultivation conditions. NUpE was highly significantly positively (*R* ^2^ > 0.8) correlated with grain yield at all the N levels and seasons (**Figure 3**). MTU 1010 (14), IR64 (11), VL Dhan 209 (9), and Heera (4) exhibited higher NUpE and grain yield at N0. MTU 1010 (14), Vasumati (10), Varadhan (13), Heera (4), Indira (5), and Birupa (2) showed maximum NUpE along with grain yield at N50. MTU 1010 (14), Vasumati (10), Varadhan (13), VL Dhan 209 (9), and Indira (5) recorded higher NUpE and grain yield at both N100 and N150 [except VL Dhan 209 (9)]. N22 (7) noted the least NUpE and grain yield at all the N levels. ANRE noted a significant (*R* ^2^ ≥ 0.5) or highly significant (*R* ^2^ ≥ 0.7) positive correlation with grain yield at all the N levels and seasons (**Figure 4**). Varadhan (13), Vasumati (10), and Indira (5) exhibited maximum ANRE along with grain yield at all the N levels whereas N22 (7) and Tella Hamsa (8) showed the least ANRE as well as grain yield. Although non-significant, NUI noted a negative relationship with grain yield at all other grades of N content in both wet and dry seasons, except for both the dry seasons at N50 and the dry season (2021) at N150 (**Figure 5**). NHI noted a significant (*R* ^2^ ≥ 0.5) or non-significant (*R* ^2^< 0.5) positive correlation with grain yield at all the N levels and seasons (**Figure 6**). Vasumati (10), Birupa (2), Indira (5), and Heera (4) noted a higher NHI along with grain yield N0 and N50 [also Varadhan (13)]. MTU 1010 (14), Vasumati (10), Varadhan (13), and Indira (5) showed higher grain yield and NHI at both N100 and N150. N22 (7) and Tella Hamsa (8) showed the least NHI along with grain yield at all the N levels.

NUtE was positively correlated with grain yield at all the N levels (**Supplementary Figure 1**). However, the correlation was significant only in the dry seasons from N50 to N150. Birupa (2), Varadhan (13), Vasumati (10), and Indira (5) have shown higher NUtE and grain yield at N50. Indira (5), VL Dhan 209 (9), Vasumati (10), and Varadhan (13) exhibited higher NUtE along with grain yield at N100 and N150 [except VL Dhan 209 (9)]. Tella Hamsa (8) noted the least NUtE along with grain yield at most of the N levels. NUE_yield_ noted a significant positive correlation (*R* ^2^ = 1) with grain yield at all N levels and seasons (**Supplementary Figure 2**). MTU 1010 (14), VL Dhan 209 (9), IR64 (11), and Heera (4) showed higher NUE_yield_ along with grain yield at N0. Vasumati (10), Birupa (2), MTU 1010 (14), Varadhan (13), and Indira (5) have exhibited maximum NUE_yield_ and grain yield at N50. At N100 and N150, MTU 1010 (14), Vasumati (10), Varadhan (13), Indira (5), and VL Dhan 209 (9) have shown higher grain yield and NUE_yield_. N22 (7) and Tella Hamsa (8) have shown the least NUE_yield_ and grain yield at all N levels. AE showed significantly positive correlation with grain yield at all N levels and seasons except for wet season 2021 at N0 (**Supplementary Figure 3**). Vasumati (10), Varadhan (13), and Birupa (2) exhibited higher AE along with grain yield at N50. At this N level, VL Dhan 209 (9) and IR64 (11) were good in grain yield and least in AE, while N22 (7) and Tella Hamsa (8) were better than VL Dhan 209 (9) and IR64 (11) in AE but poor in grain yield. At N100 and N150, Varadhan (13), MTU 1010 (14), Vasumati (10), and Indira (5) noted maximum AE and grain yield whereas N22(7) and Tella Hamsa (8) noted the least AE and grain yield. PE noted a non-significant positive correlation with grain yield at all N levels in most of the seasons while it noted a non-significant negative correlation at N100 and N150 levels in wet season 2020 (**Supplementary Figure 4**). MTU 1010 noted higher PE along with grain yield at all N levels. Moreover, all the tested genotypes noted similar PE values at both N100 and N150 and differed in yield. PFP noted a highly significant positive correlation (*R* ^2^ = 1) with grain yield at all N levels and seasons (**Supplementary Figure 5**). Vasumati (10), Varadhan (13), MTU 1010 (14), and Indira (5) noted higher PFP along with grain yield at all the N levels whereas N22 (7) and Tella Hamsa (8) were the least.

## Discussion

Nitrogen (N) is an essential nutrient for the growth, development, and maintenance of rice (Wang et al., 2022). As soil N fertilizer alone is not adequate for increase in rice production, farmers add higher amounts of N fertilizer expecting that increased application of N fertilizer will result in the enhanced yields (West et al., 2014; Wang et al., 2022). Higher N fertilizer inputs are leading to serious environmental problems and low production efficiency (Wang et al., 2022). As only 30 to 50% of applied N is reported to be utilized by rice, reduction of N fertilizer application by 50% of the recommended N was chosen as the current target for NUE in rice (Ladha et al., 2020). Variation in rice varietal response to graded N application, especially 50% of recommended N, has been studied (Singh et al., 1998; Singh et al., 2014; Vijayalakshmi et al., 2015). Application of N fertilizer without considering the NUE of a particular variety leads to not only reduced use efficiency but also environmental pollution and increased cost of cultivation. In the present study, to evaluate the genotype response with varying yield potential to graded N application, 14 rice genotypes were assessed at four levels of N fertilizer. To identify physiological traits associated with grain yield in selecting promising genotypes at reduced N application (50% of the recommended N), flag leaf characteristics (including N content), photosynthetic pigment content, gas exchange traits, and chlorophyll fluorescence characteristics were studied. In addition, NUE indices were also estimated for their suitability to select N efficient genotypes under reduced N application.

**Figure 7:**
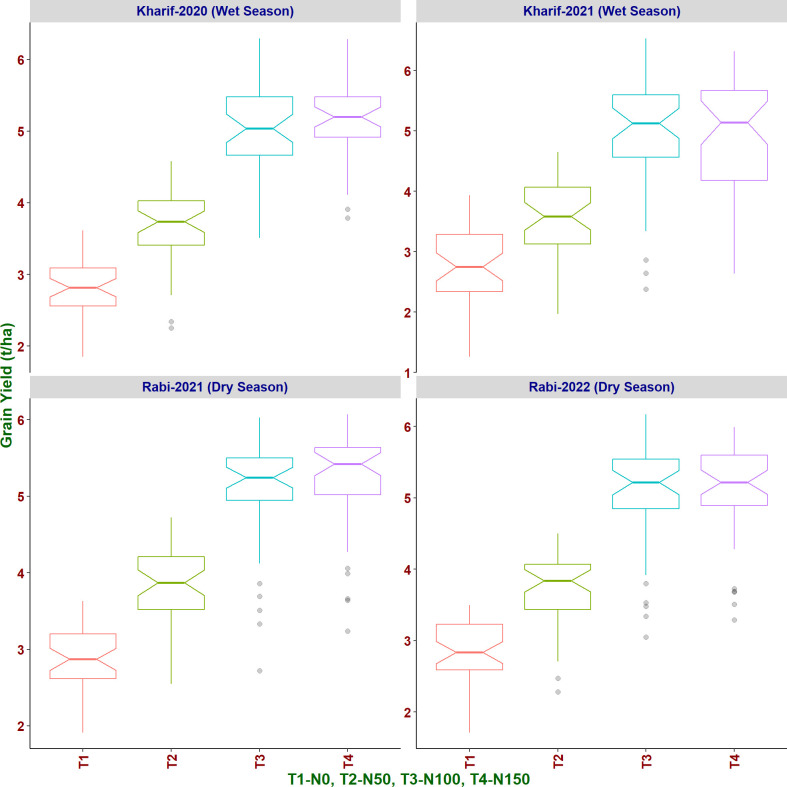
Effect of increased levels of N application on grain yield of rice genotypes in different seasons.

Under N50, a reduction of 26.99% of grain yield in comparison with N100 was observed while 43.88% reduction was observed under N0. However, only marginal increment in grain yield (1.31%) was observed from N100 to N150 (**Figure 7**). Birupa exhibited the least reduction (15.31%) in grain yield at N50 compared to N100 whereas Daya exhibited the highest reduction (34.20%). Genotypic differences were earlier reported for grain yield of rice at different N levels (Singh et al., 1998). Thus, to achieve the reduction of N fertilizer application, the selection of genotypes is crucial because of their inherent response for N. With the increased N application, previous studies also reported increase in grain yield, which is attributed to increased tillering, number of panicles, and grains (Devika et al., 2018; Zhang et al., 2020; Bama et al., 2021; Karmakar et al., 2021; Liang et al., 2021; Xin et al., 2022; Zhu et al., 2022) and increase in total dry matter accumulation (Pan et al., 2012; Singh et al., 2014; Jyothi Swaroopa and Lakshmi, 2015; Zhang et al., 2020). Days to 50% flowering and days to physiological maturity also increased significantly with increased N application due to increased vegetative growth phase (Mahajan et al., 2011; Rajesh et al., 2017; Wani et al., 2017; Ghoneim and Osman, 2018; Bv et al., 2019; Ye et al., 2019b; Mandal et al., 2022) and increased tillering (Wang et al., 2016).

As expected, six flag leaf traits (FLL, FLW, FLA, FLT, FLDW, and SLA) have shown a significant increase and specific leaf weight (SLW) has shown a significant decrease with increased N application in the present study. Earlier studies have also reported a significant increase in length and width of flag leaf (Bahmaniar and Ranjbar, 2007), an increase in leaf area and leaf thickness with increased N application (Vijayalakshmi et al., 2015), a significant increase in leaf thickness from 0.31 mm at N0 to 0.54 mm at N150 (Devika et al., 2018), and a significant and the highest increase in leaf thickness and leaf dry mass at N270 (Hou et al., 2020) in rice. Similarly, reduction in SLW of rice with increased N application (Yang et al., 2003; Huang et al., 2008), under sufficient N compared to low N treatment in inbred *indica* rice cultivars (Liu and Li, 2016) and 2.9% to 11.1% reduction as the N application levels gradually increased from N0 to N270 (Hou et al., 2020), was reported, supporting our observations. Likewise, SLA increase was also reported with increase in N application at crown root initiation stage in wheat (Alam, 2014).

In congruence with our results, the photosynthetic pigment contents were elevated (Jinwen et al., 2009; Cisse et al., 2020; Hou et al., 2020) or showed an upward trend in *indica* hybrid rice (Peng et al., 2021) and *japonica* rice (Gong et al., 2022) with the increase in N application rates. Appropriate N application was shown to improve the enzyme and chlorophyll content of plant leaves, thereby improving the photosynthetic activities of the plant (Giersch and Robinson, 1987; Nduwimana, 2020).

Pn and E are the crucial physiological processes for NUE and Pn was significantly higher for the higher NUE genotypes, relative to the lower NUE genotypes (Kumari et al., 2021). Increased amounts of nitrate supply significantly enhanced Pn, g_s_, and E (Mandal et al., 2022). As noted in this study, with the increase in N application level from 0 to 200 kg ha^–1^, Pn, g_s_, and E were also increased gradually, while C_i_ values were decreased (Gong et al., 2022). Increased N application increased the Pn that noted a positive correlation with leaf N content (Fallah, 2012; Rajesh et al., 2017; Bv et al., 2019; Zhang et al., 2020), increased the E (Zhang et al., 2020), and increased the g_s_ at the vegetative stage (Roy Chowdhury et al., 2014). Significantly higher values were recorded for g_s_ and E with N100 compared to N0 (Vijayalakshmi et al., 2015). Compared with low N (0 kg N ha^−1^), Pn, g_s_, and E were significantly higher under medium (120 kg N ha^−1^) and high N (180 kg N ha^−1^) levels (Pan et al., 2016). A significantly higher Pn of 29.52 µmol (CO_2_) m^−2^ s^−1^ at 150% of RDN was noted compared with a Pn of 17.41 µmol (CO_2_) m^−2^ s^−1^ at 0% of RDN (Devika et al., 2018). Flag leaf N content increased significantly with increased N application and is in accordance with the earlier findings (Swarna et al., 2017; Cisse et al., 2020; Hou et al., 2020). Leaf N plays a crucial role in photosynthesis, which ultimately affects biomass production (Ladha et al., 1998). PNUE is the photosynthetic capacity per unit leaf N. PNUE is a key component of NUE and an indicator of the relationship between leaf N and Pn. In the current investigation, PNUE increased with an increase in rate of N application. The higher the PNUE, the higher the crop N utilization rate (Mugo et al., 2021). Leaf N allocation is an important factor influencing PNUE. Suitable N application can improve the leaf photosynthetic rate, which helps to increase the PNUE, which, in turn, enhances the crop yield (Zhao et al., 2013).

Among the chlorophyll fluorescence traits, F_v_/F_m_, ΦPSII, ETR, and qP showed an increasing trend, while qN decreased as the N rate increased as reported in hybrid rice (Peng et al., 2021). The application of the appropriate amount of N could increase the solar energy conversion efficiency in the PSII reaction center of rice leaves by improving the electron transfer efficiency and enhancing electron flow (Zhang et al., 2017; Fu et al., 2021). Among the total traits of the study, in comparison with N100, most of the chlorophyll fluorescence traits (F_v_/F_m_, ΦPSII, ETR, and qP) and C_i_ among the gas exchange traits were significantly correlated with grain yield at N50. As these traits were measured using flag leaf, the correlation of these traits with FLN revealed significant differences between N50 and N100. Hence, flag leaf at 50% flowering can be a good source to assess chlorophyll fluorescence traits under reduced N conditions and can differentiate rice genotypes varying in yield and NUE. In photosystem II of light reaction, F_v_/F_m_ explains the maximum photochemical conversion (quantum yield), ΦPSII explains the effective photochemical conversion, ETR explains the amount of electron transfer at the reaction center in PSII, and qP represents the functional or open proportion of PSII involved in photochemical conversion. The available literature indicates that N-deficient conditions like N50 can result in improper formation and function of PSII affecting photochemical conversion followed by yield (Jin et al., 2012) and reduce carboxylation efficiency (Huang et al., 2004), whereas proper or optimum availability of N improves the function of PSII, quantum efficiency, and grain yield (Liu and Xu, 2018). Hence, results obtained in the current investigation suggest the usage of these physiological traits (F_v_/F_m_, ΦPSII, ETR, qP, and C_i_) to screen genotypes under N50 with known low-yielding and high-yielding genotypes as checks. As optimum N can show the highest quantum efficiency followed by yield and genotype-specific optimum N requirement is unknown, Birupa, a moderate yielder at N100, emerged as one of the top five yielders at N50 and *vice versa* in the case of VL Dhan 209. It indicates differential response of the genotypes for yield potential with varied levels of N.

Increase in grain, straw, and total N uptake with increased N application as observed in the present study is in concurrence with the earlier findings (Tayefe et al., 2011; Swarna et al., 2017; Bama et al., 2021). AE, PE, ANRE, and PFP are indices for NUE proposed by Dobermann (2007). AE represents the contribution of fertilizer N towards yield in comparison to a non-fertilized control and is helpful to assess the contribution of added N fertilizer in enhancing the yield. PE represents the contribution of fertilizer N from the plant tissues to increase yield and is useful to identify plants that have a superior ability in producing yield per unit of available N. ANRE is the percentage of fertilizer N that is taken up by the plant and it aids in studying crop response to the applied N fertilizer. Both PE and ANRE account for background (available) soil N (Congreves et al., 2021). N application rate showed a significant effect on AE, PE, and ANRE and was maximum with N100 followed by N50 and minimum with N150. Among the treatments, cumulative mean values of AE ranged from 15.1 at N150 to 22.0 at N100, PE ranged from 41.5 at N150 to 46.1 at N100, and ANRE ranged from 36.8 at N150 to 48.5 at N100. AE and ANRE of rice were decreased with increasing N application over N100 and indicated that the capability of increase in yield per kilogram of applied N declined remarkably with increasing N application greater than N100. PE also decreased with increasing N application over N100 and showed that yield increased per kilogram N accumulated in rice plant was decreased with increasing N application greater than N100. AE was 16–36 in Boro rice (Islam et al., 2015) and 0.52–17 in T. Aman rice (Hussain et al., 2016). AE significantly increased with increasing N levels up to 165 kg N ha^−1^ and decreased with further uplift in N application in some recently released Egyptian rice varieties (Ghoneim and Osman, 2018). AE decreased at N120 and N150 (Mboyerwa et al., 2022). Similarly, ANRE increased at first, reached the maximum under optimum N application, and thereafter declined significantly under higher N levels (Ye et al., 2007) and at N160 (Katuwal et al., 2021). PE also decreased significantly at N150 (Kumar et al., 2015). The diminishing trend of PE at higher N rates pointed out that rice plants are unable to absorb or utilize N at higher rates of N application or the rate of N uptake by plant cannot keep pace with the loss of N. AE, PE, and ANRE decreased gradually with an increase in N rate from 3.5 to 14 g m^−2^ in nerica-4 (Yesuf and Balcha, 2014). Partial factor productivity (PFP) is the simplest form of NUE efficiency and is calculated as yield per applied N. It is a convenient index for comparing management practices on a single crop type. Mean values of PFP decreased significantly with increase in N application from 73.3 at N0 to 33.9 at N150 in the present study and is in accordance with previous findings (Pan et al., 2017; Rea et al., 2019). Similar results were also reported by other researchers in their studies (Ye et al., 2007; Cheng et al., 2011; Tayefe et al., 2011).

NUpE is the percentage of available soil N taken up by the plant (Moll et al., 1982) and is useful for sustainable cultivation of rice. The cumulative mean NUpE values increased with an increase in N application from 28.8 at N0 to 32.2 at N100 and declined to 29.0 at N150. NUtE is the contribution of plant N towards yield (Moll et al., 1982). NUtE decreased with increased N application and mean NUtE ranged from 51.5 (N0) to 46.2 (N150). Similarly, higher NUtE was recorded at 0% RDN, and the lower value was recorded at 150% RDN (Devika et al., 2018). Three rice cultivars with similar growth periods tested under different N levels had dissimilar grain yield, N absorption, and utilization rates (Xin et al., 2022). They also found that at low N, rice yield was mainly limited by NUpE, while at high N, yield was mainly limited by NUtE. Increased flag leaf N content and delayed leaf senescence could improve NUtE (Vijayalakshmi et al., 2013). Hence, to maintain stable grain yield at different N levels, both N uptake and utilization efficiencies should be simultaneously improved. In low N conditions, NUpE is more important than NUtE (Witcombe et al., 2008; Khan et al., 2017). NUE_yield_ indicates the contribution of available N towards grain yield (Novoa and Loomis, 1981) and enables comparison of yield potential among genotypes. Among the treatments, mean NUE_yield_ increased from 14.7 at N0 to 15.6 at N100 and decreased to 13.4 at N150. It is indicated that NUE_yield_ did not increase linearly with the amount of N application (Kunta and Thatikunta, 2020). Likewise, NUE_yield_ increased up to 100% RDN and decreased with a further increase in N levels up to 150% RDN and also concluded that the application of excess N was not effectively utilized by the crop and the production rate per unit of N applied was low (Kumar et al., 2008). Lower PE under high N supply results in lower NUE_yield_ (Li et al., 2012). With the increase in N application rates (0, 160, 210, 260, 315, and 420 kg N ha^−1^), NUE_yield_ increased up to 210 kg N ha^−1^ and then decreased (Liang et al., 2021). NUI is the contribution of plant N towards accumulation of plant biomass (Huang et al., 2018). NUI decreased significantly with increased application of N and mean NUI decreased from 136.1 at N0 to 99.3 at N150. NHI is the amount of plant N present in the yield component (grain in the case of rice) (Moll et al., 1982) and can be used to identify plants with greater N translocation efficiency to the economic part. NHI increased with increase in N application rate from N0 (55.4) up to N100 (63.2) and slightly decreased with N150 (62.5). The increase in NHI up to N100 may be due to the increase in grain yield, and the transfer of N to the grain is greater than the increase in total plant N. Although NHI of rice decreased with increasing N application over N100, N ratio in straw enhanced over grain. NHI may be useful in selecting crop genotypes for higher grain yield (Fageria and Baligar, 2005). Out of nine NUE indices assessed in 14 genotypes under graded N levels, NUpE, NUtE, and NUE_yield_ delineated the best-performing genotypes under N50.

Screening of 14 genotypes under four graded N levels across four seasons revealed wide genotypic variation in their response in terms of grain yield. An increase of agro-morphological traits, photosynthetic pigments, and flag leaf traits (except SLW) was observed with an increase of N fertilizer application. At N50, F_v_/F_m_, ΦPSII, ETR, qP, and C_i_ of flag leaf at flowering noted significant association with grain yield. Of the 14 genotypes, the top 5 (MTU 1010, Indira, Varadhan, VL Dhan 209, and Vasumati) grain yielders at N100 were identified as promising genotypes for efficient use of N by NUpE, NUtE, and NUE_yield_ indices at N50. Moreover, NUE_yield_ is the product of NUpE and NUtE. Hence, among the nine indices, these three (NUpE, NUtE, and NUE_yield_) can be further used to identify promising genotypes at N50.

## Conclusion

The present study has clearly demonstrated the existence of genetic variability among the rice genotypes through N response under graded N levels. The grain yield penalty ranged only from 15% to 34.2% at N50 across the 14 genotypes in comparison with N100, suggesting the possibility of reduction of N fertilizer application. Most importantly, through the evaluation of flag leaf physiological traits at the flowering stage, chlorophyll fluorescence traits (F_v_/F_m_, ΦPSII, ETR, and qP) and C_i_ were identified to be associated with grain yield under N50, which could be deployed in the breeding for NUE in rice. Among the tested genotypes, Birupa, which is a relative moderate yielder at N100, emerged as a high yielder under N50, which indicates the potential of the moderate-yielding genotypes at N100 to produce better grain yield at N50. Therefore, this study recommends the evaluation of the released rice varieties at N50 to determine their suitability under low N input conditions. Among the nine NUE indices studied, NUpE, NUtE, and NUE_yield_ are useful to identify promising genotypes at N50.

## Data availability statement

The original contributions presented in the study are included in the article/**Supplementary Material**. Further inquiries can be directed to the corresponding author.

## Author contributions

BS: Data curation, Formal analysis, Investigation, Methodology, Writing – original draft. DS: Conceptualization, Funding acquisition, Project administration, Resources, Supervision, Writing – review & editing. DSR: Data curation, Software, Visualization, Writing – review & editing. SR: Methodology, Writing – review & editing. KaS: Software, Writing – review & editing. PR: Resources, Writing – review & editing. KuS: Methodology, Writing – review & editing. RS: Resources, Writing – review & editing. CN: Conceptualization, Supervision, Writing – review & editing.

## Supplementary material

Supplementary Figure 1 Relationship between nitrogen utilization efficiency (NUtE) and grain yield of rice genotypes at different N levels and seasons. 1, Anjali; 2, Birupa; 3, Daya; 4, Heera; 5, Indira; 6, Nidhi; 7, N22; 8, Tella Hamsa; 9, VL Dhan 209; 10, Vasumati; 11, IR64; 12, GQ25; 13, Varadhan and 14, MTU 1010.

Supplementary Figure 2 Relationship between nitrogen use efficiency_yield_ (NUE_yield_) and grain yield of rice genotypes at different N levels and seasons. 1, Anjali; 2, Birupa; 3, Daya; 4, Heera; 5, Indira; 6, Nidhi; 7, N22; 8, Tella Hamsa; 9, VL Dhan 209; 10, Vasumati; 11, IR64; 12, GQ25; 13, Varadhan and 14, MTU 1010.

Supplementary Figure 3 Relationship between agronomic efficiency (AE) and grain yield of rice genotypes at different N levels and seasons. 1, Anjali; 2, Birupa; 3, Daya; 4, Heera; 5, Indira; 6, Nidhi; 7, N22; 8, Tella Hamsa; 9, VL Dhan 209; 10, Vasumati; 11, IR64; 12, GQ25; 13, Varadhan and 14, MTU 1010.

Supplementary Figure 4 Relationship between physiological efficiency (PE) and grain yield of rice genotypes at different N levels and seasons. 1, Anjali; 2, Birupa; 3, Daya; 4, Heera; 5, Indira; 6, Nidhi; 7, N22; 8, Tella Hamsa; 9, VL Dhan 209; 10, Vasumati; 11, IR64; 12, GQ25; 13, Varadhan and 14, MTU 1010.

Supplementary Figure 5 Relationship between partial factor productivity (PFP) and grain yield of rice genotypes at different N levels and seasons. 1, Anjali; 2, Birupa; 3, Daya; 4, Heera; 5, Indira; 6, Nidhi; 7, N22; 8, Tella Hamsa; 9, VL Dhan 209; 10, Vasumati; 11, IR64; 12, GQ25; 13, Varadhan and 14, MTU 1010.
