# Supplementary figures and images for: Promising physiological traits associated with nitrogen use efficiency in rice under reduced N application
Source: Front Plant Sci. 2023 Nov 20;14:1268739. doi: 10.3389/fpls.2023.1268739 (PMC10694615; doi:10.3389/fpls.2023.1268739)

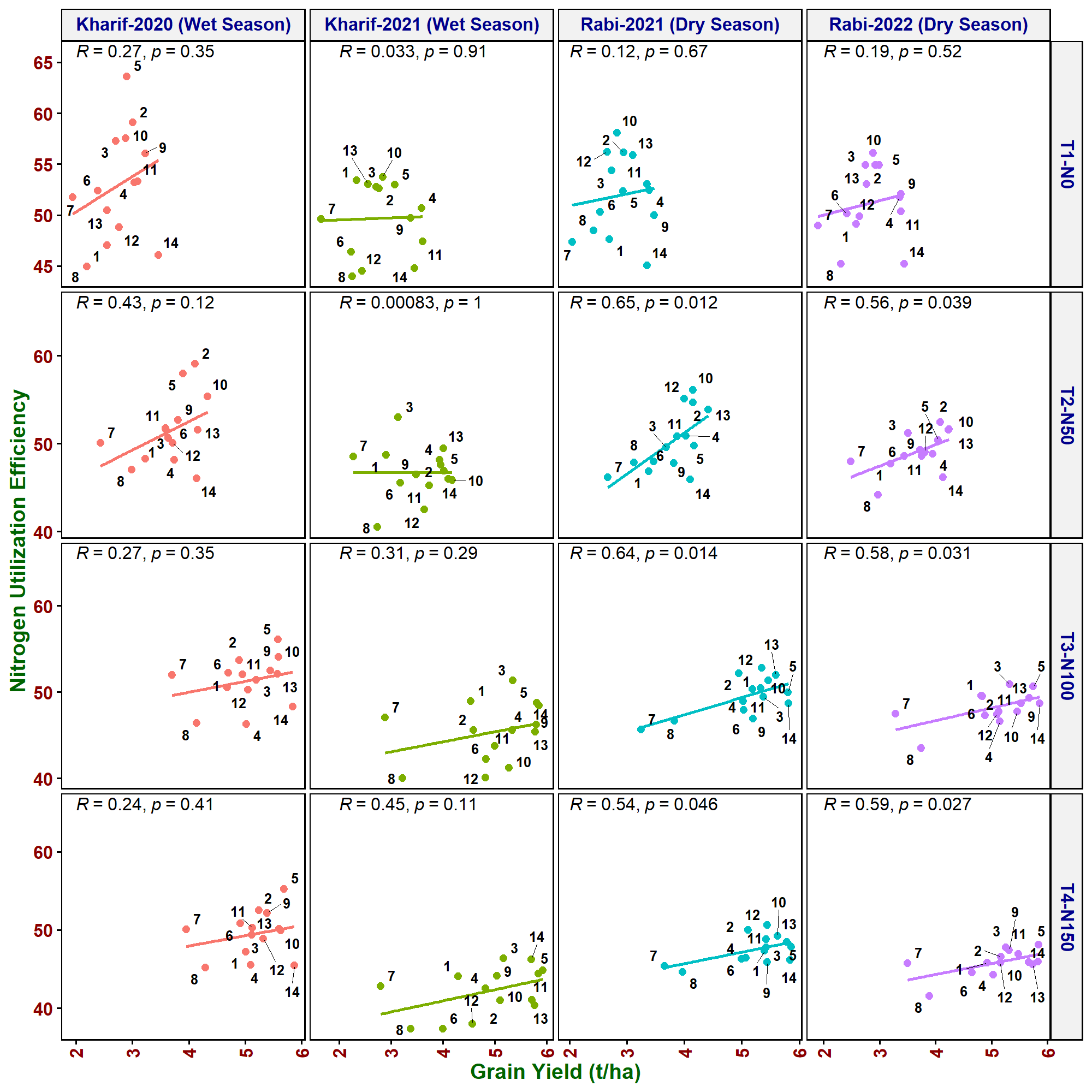

Supplement: Supplementary Figure 1 — Relationship between nitrogen utilization efficiency (NUtE) and grain yield of rice genotypes at different N levels and seasons. 1, Anjali; 2, Birupa; 3, Daya; 4, Heera; 5, Indira; 6, Nidhi; 7, N22; 8, Tella Hamsa; 9, VL Dhan 209; 10, Vasumati; 11, IR64; 12, GQ25; 13, Varadhan and 14, MTU 1010. [file Image_1.tiff]

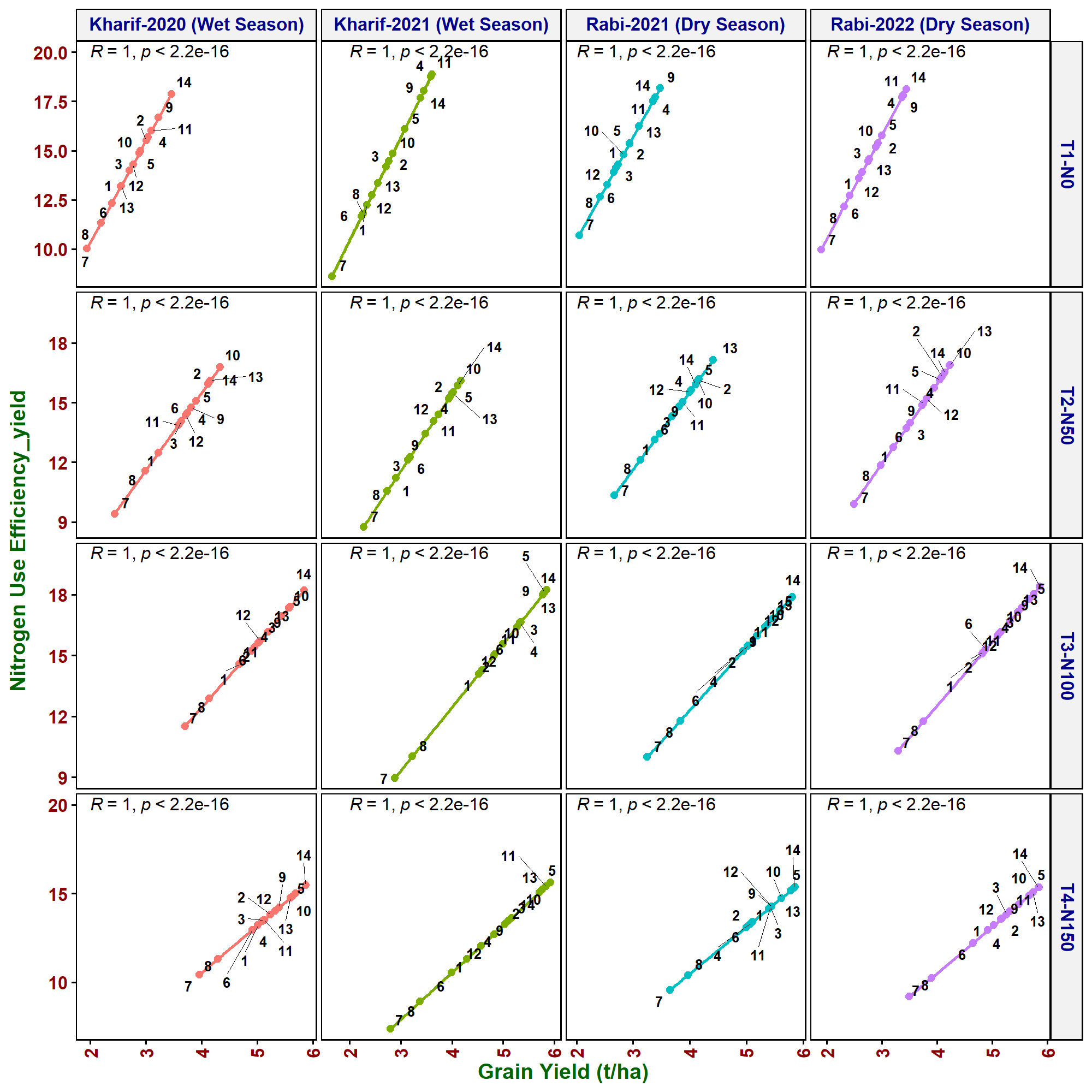

Supplement: Supplementary Figure 2 — Relationship between nitrogen use efficiencyyield (NUEyield) and grain yield of rice genotypes at different N levels and seasons. 1, Anjali; 2, Birupa; 3, Daya; 4, Heera; 5, Indira; 6, Nidhi; 7, N22; 8, Tella Hamsa; 9, VL Dhan 209; 10, Vasumati; 11, IR64; 12, GQ25; 13, Varadhan and 14, MTU 1010. [file Image_2.tiff]

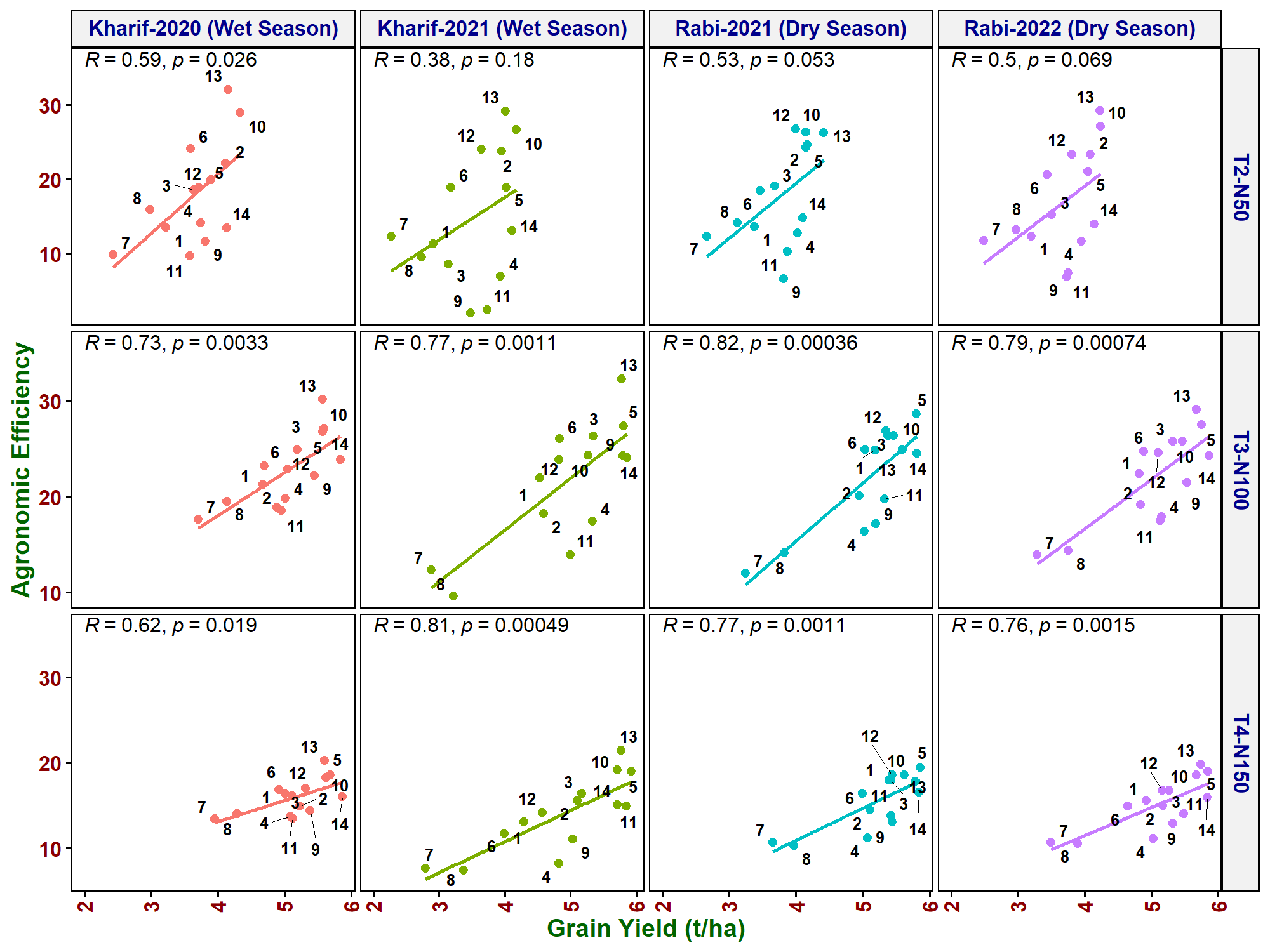

Supplement: Supplementary Figure 3 — Relationship between agronomic efficiency (AE) and grain yield of rice genotypes at different N levels and seasons. 1, Anjali; 2, Birupa; 3, Daya; 4, Heera; 5, Indira; 6, Nidhi; 7, N22; 8, Tella Hamsa; 9, VL Dhan 209; 10, Vasumati; 11, IR64; 12, GQ25; 13, Varadhan and 14, MTU 1010. [file Image_3.tiff]

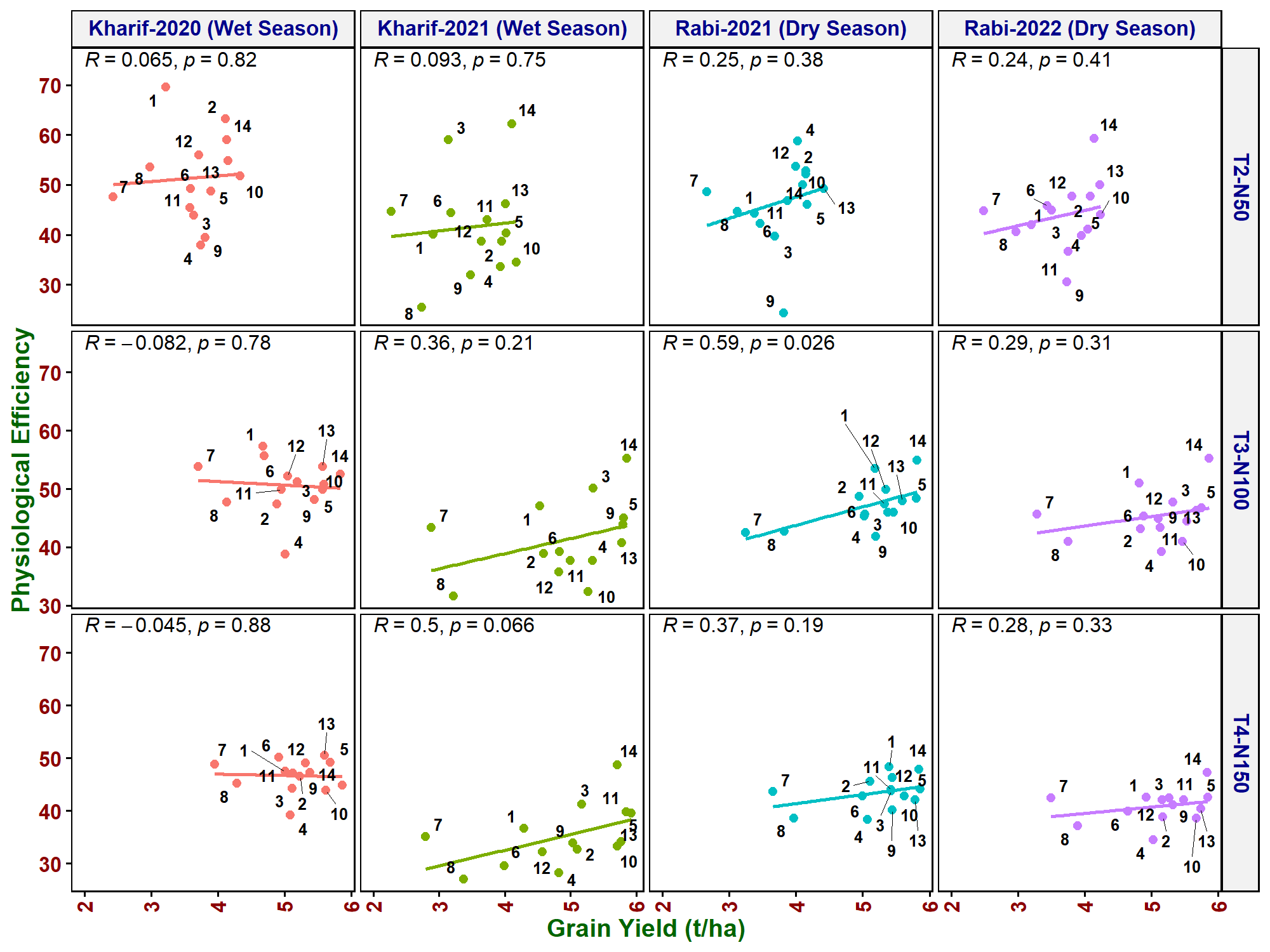

Supplement: Supplementary Figure 4 — Relationship between physiological efficiency (PE) and grain yield of rice genotypes at different N levels and seasons. 1, Anjali; 2, Birupa; 3, Daya; 4, Heera; 5, Indira; 6, Nidhi; 7, N22; 8, Tella Hamsa; 9, VL Dhan 209; 10, Vasumati; 11, IR64; 12, GQ25; 13, Varadhan and 14, MTU 1010. [file Image_4.tiff]

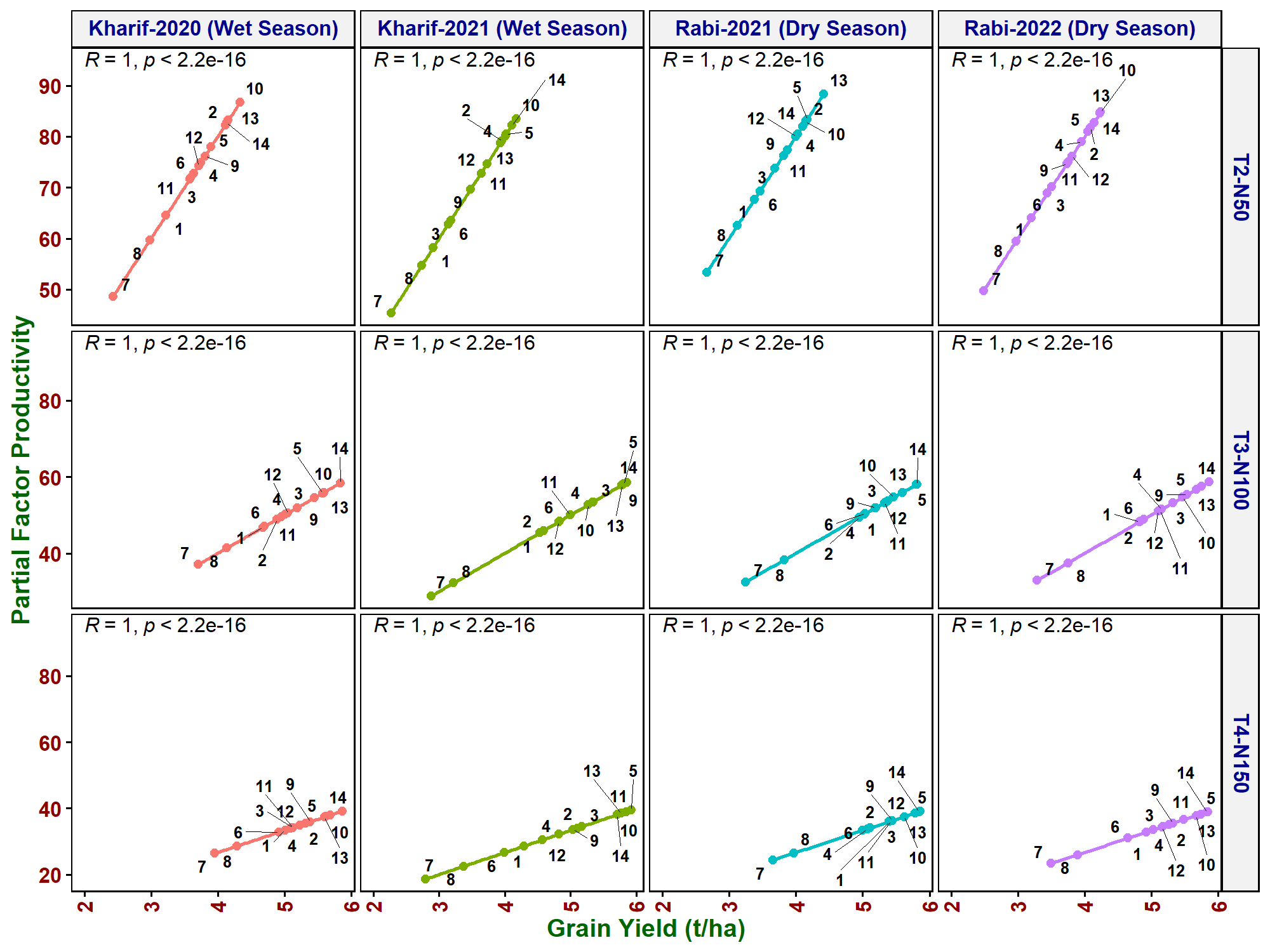

Supplement: Supplementary Figure 5 — Relationship between partial factor productivity (PFP) and grain yield of rice genotypes at different N levels and seasons. 1, Anjali; 2, Birupa; 3, Daya; 4, Heera; 5, Indira; 6, Nidhi; 7, N22; 8, Tella Hamsa; 9, VL Dhan 209; 10, Vasumati; 11, IR64; 12, GQ25; 13, Varadhan and 14, MTU 1010. [file Image_5.tiff]
